# Recent Advancements in Molecular Therapeutics for Corneal Scar Treatment

**DOI:** 10.3390/cells11203310

**Published:** 2022-10-21

**Authors:** Anwesha Ghosh, Vijay K. Singh, Vivek Singh, Sayan Basu, Falguni Pati

**Affiliations:** 1 Department of Biomedical Engineering, Indian Institute of Technology Hyderabad, Kandi, Sangareddy 502284, Telangana, India; 2 SSR-Stem Cell Biology Laboratory, Center for Regenerative Ophthalmology, L V Prasad Eye Institute, Hyderabad 500034, Telangana, India; 3 Centre for Ocular Regeneration (CORE), L.V. Prasad Eye Institute, Hyderabad 500034, Telangana, India

**Keywords:** corneal scar, molecular therapeutics, exosome, si/microRNAs, recombinant viral vectors, bioactive molecules, nanotechnology

## Abstract

The process of corneal wound healing is complex and induces scar formation. Corneal scarring is a leading cause of blindness worldwide. The fibrotic healing of a major ocular wound disrupts the highly organized fibrillar collagen arrangement of the corneal stroma, rendering it opaque. The process of regaining this organized extracellular matrix (ECM) arrangement of the stromal layer to restore corneal transparency is complicated. The surface retention capacity of ocular drugs is poor, and there is a large gap between suitable corneal donors and clinical requirements. Therefore, a more efficient way of treating corneal scarring is needed. The eight major classes of interventions targeted as therapeutic tools for healing scarred corneas include those based on exosomes, targeted gene therapy, microRNAs, recombinant viral vectors, histone deacetylase inhibitors, bioactive molecules, growth factors, and nanotechnology. This review highlights the recent advancements in molecular therapeutics to restore a cornea without scarring. It also provides a scope to overcome the limitations of present studies and perform robust clinical research using these strategies.

## 1. Introduction 

**Figure 1:**
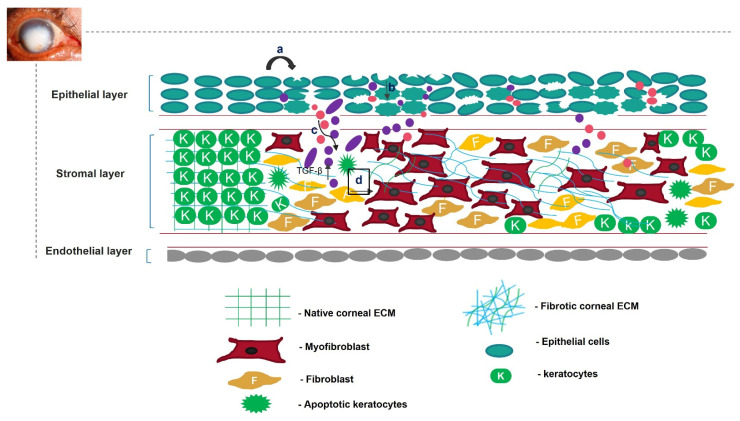
Overview of corneal wound-healing process and scar formation. After an ocular wound, specifically epithelial wounding, (**a**) the epithelial cells undergo apoptosis, (**b**) and epithelial cell markers are lost because of corneal epithelial injury, triggering their migration and proliferation. A deep injury disrupts Bowman’s membrane, allowing the invasion of neutrophils (pink) and macrophages (purple) into the stromal layer and releasing TGF-β. This increases the concentration of TGF-β in the stromal layer (**c**). Therefore, the keratocytes undergo apoptosis, differentiate into myofibroblasts, and disrupt the arrangement of the corneal ECM (**d**).

Unregulated tissue fibrosis is generally associated with the development of chronic diseases. Ocular insults to the cornea, such as alkali burns, injuries, and inflammation, trigger the complex process of wound healing to maintain its structural integrity, functional integrity, and transparency [1]. This is mainly characterized by the migration and proliferation of corneal epithelial cells, followed by the differentiation of stromal keratocytes into myofibroblasts, the excess deposition of extracellular matrix (ECM) components, an increase in alpha-smooth muscle actin (α-SMA) expression, and ECM remodeling, resulting in corneal opacity [2,3]. The accumulation of these events leads to a scarred cornea (Figure 1). Corneal scarring is the fourth most common cause of blindness, affecting approximately five million people globally each year [4]. A scarred cornea is classified according to its degree of opacity. Nebular opacity is caused by superficial scars on the ocular surface. As the severity of corneal scarring increases, the degree of opacity progresses from nebular to macular and leucoma [5]. Superficial scars heal over a period of three months, restoring vision; however, deep scars generally worsen, leading to blindness [6].

Finding an appropriate therapeutic technique to heal a scarred cornea efficiently has proven difficult. Corneal transplantation is an unsuitable therapy for treating scarring because of the lack of appropriate corneal donors [7]. The use of the amniotic membrane as an ocular bandage because of its anti-angiogenic and antifibrotic properties is a popular treatment method. However, its use is limited because of ethical problems related to tissue donation, potential risks of the transmission of diseases, inter-amnion variability, and reproducibility [8]. Topical ocular drops are a simple way of treating a scarred cornea. However, their low bioavailability and rapid drainage from the ocular surface limit their popularity [9]. Therefore, there is a need for a therapeutic method that can effectively harness the body’s signaling cascade to halt fibrosis and heal the corneal wound.

This review highlights the recent advancements in therapeutic approaches to treating a scarred cornea at the molecular level. The following eight classes of interventions have been targeted as therapeutic tools for treating scarred cornea: exosomes, microRNAs (miRNAs), recombinant viral vectors, bioactive molecules, growth factors, histone deacetylase inhibitors, and nanotechnology. Exosomes can be used as delivery vehicles for transporting therapeutic cargo, such as antifibrotic miRNAs or proteins from stem cells to the target cells. Small interfering RNAs (siRNAs) or miRNAs can be used to silence the expression of specific fibrotic genes and treat corneal scarring. A scarred cornea can also be treated by modulating the levels of growth factors, such as insulin-like growth factor 1 (IGF-1) and transforming growth factor beta (TGF-β), which are critical for fibrosis. Epigenetic modulators, such as histone deacetylase inhibitors, or clinically accepted biomolecules, such as chitosan, glucosamine, *Lycium barbarum* polysaccharide, decorin, TPCA-1, or acetylcholine, are recent medicaments that can heal a scarred cornea via their respective biological mechanisms. This review also highlights the advances in nanotechnology for corneal treatment. Understanding the limitations of the existing corneal scarring remedies might provide insights for developing new therapeutic modules to restore vision in scarred-cornea patients.

## 2. Scarring of Cornea

The cornea is a structurally and functionally integrated avascular organ with three cellular layers interrupted by two acellular layers [10]. It is resistant to minor wounds; however, major ocular trauma can compromise its transparency [11]. A significant abrasion might disrupt the basement and Bowman’s membranes, leading to interactions between the epithelial and stromal layers of the cornea. This interaction initiates the complex wound-healing process [12,13]. Various growth factors, such as fibroblast growth factor (FGF), epidermal growth factor (EGF), and IGF-1, cross the disrupted Bowman’s membrane from the tear film to reach the stromal layer of the cornea during the initial wound-healing process [14,15]. In the epithelial layer, wounded epithelial cells undergo apoptosis, migration, and proliferation. The concentration of interleukin-1 (IL-1) increases in the epithelial layer upon injury. Therefore, IL-1 crosses the disrupted Bowman’s membrane into the stromal layer and becomes a major factor in killing keratocytes [16].

In the stromal layer, the corneal injury increases the expression of keratocyte membrane-bound αVβ/αβ integrins [17] and various metalloproteases, such as matrix metalloprotease 2 (MMP2) or MMP9 [18]. This cumulatively activates TGF-β from latency [19,20]. Neutrophils, monocytes, and macrophages infiltrate the wounded area during the healing process. They either become incorporated into fibrous DNA-like structures known as neutrophil extracellular traps of ECM or secrete TGF-β, increasing the concentration of TGF-β in the stromal layer [21,22,23,24]. Therefore, TGF-β takes over the control of the corneal wound-healing process. Quiescent keratocytes transdifferentiate into myofibroblasts, enlarged spindle-shaped cells characterized by the neo-expression of α-SMA in the presence of TGF-β. Incorporating α-SMA into the corneal ECM generates a contractile force that disrupts the orthogonal arrangement of its collagen fibrils, altering its normal curvature [25,26].

**Figure 2:**
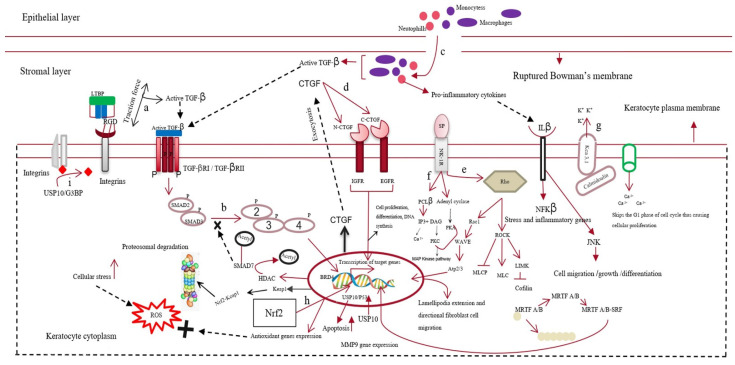
Mechanism of corneal scarring. This figure represents the keratocytes of the stromal layer, the epithelial layer, the disrupted Bowman’s membrane, and the stromal layer of the human cornea. The mechanism of corneal scarring is depicted in a single keratocyte cell for simplicity; (**a**) activation of transforming growth factor-β (TGF-β) from its latent state; (**b**) SMAD-dependent TGF-β signaling pathway; (**c**) infiltration of neutrophils and macrophages into the stromal layer and their secretion of active TGF-β; (**d**) CTGF (connective tissue growth factor) signaling pathway; (**e**) substance-P-mediated activation of the Rho/ROCK signaling cascade, leading to actin polymerization and MMP gene expression; (**f**) increase in intracellular Ca level by Substance-P-mediated PCL-β activation; (**g**) Kca3.1 ion-channel-mediated hyperpolarization of the keratocyte plasma membrane and subsequent influx of Ca ions; (**h**) Nrf2 (nuclear factor erythroid 2–related factor 2)-mediated antioxidant gene production; however, BRD4 (bromodomain-containing protein 4) increases keap1 expression, leading to the formation of the kelch-like ECH-associated protein 1 (keap1)/Nrf2 complex and their subsequent degradation; (**i**) deubiquitylation of integrins by the USP10/G3BP complex.

The active form of TGF-β first binds to the keratocyte plasma-membrane-bound TGF-β type I/II receptor (TBRI/II) and switches on the downstream SMAD-dependent signaling cascade [27]. This increases the expression of various fibrotic genes, such as MMPs and connective tissue growth factor (CTGF) [28]. In addition to TGF-β, other neuropeptides, such as calcitonin gene-related peptide, vasoactive intestinal peptide, and substance P (SP), are released from the axonal nerve endings of corneal epithelial layers. These neuropeptides cross the disrupted Bowman’s membrane to reach the stromal layer of the cornea. Substance P binds to the neurokinin 1 receptor (NK1R) of keratocytes and activates three different signaling pathways [29]. The SP/NK1R complex activates the RhoA/ROCK pathway to phosphorylate LIM kinase (LIMK). This inactivates cofilin (an actin-cleaving enzyme) [30], preventing actin degradation and promoting actin polymerization. Actin mostly remains bound to myocardin-related transcription factors A/B (MRTFA/B); however, the increased actin polymerization allows myocardin-related transcription factor A/B (MRTFA/B) to bind to serum response factor, activating profibrotic genes, such as MMPs [31,32]. The SP/NK-IR complex activates phospholipase C-beta and adenylyl cyclase, increasing the intracellular calcium level and cyclic adenosine monophosphate (cAMP) concentrations. This switches on protein kinase A and activates the extracellular signal-regulated kinase/mitogen-activated protein kinase (ERK/MAPK) pathway, increasing the proliferation of keratocytes and fibroblast cells [33,34]. The increased Ca^2+^ ions bind to calmodulin and cohere with the Kca3.1 ion channel of keratocytes to hyperpolarize the cells. This causes the cells to skip the G1 phase of the cell cycle, promoting fibrotic cell proliferation [35]. The increase in cellular stress increases the intracellular reactive oxygen species (ROS) levels, which share a positive feedback loop with the secretion of various growth factors, such as TGF-β, proinflammatory cytokines, etc. [36] Interleukin 10 (IL-10), a proinflammatory cytokine, binds to its cognate receptor and activates the nuclear factor kappa B (NF-κβ) pathway, increasing stress and other inflammatory genes’ expression [37]. TGF-β also increases the expression of bromodomain-containing protein 4 (BRD4), increasing the expression of kelch-like ECH-associated protein 1 (keap1). Keap1 might bind to the antioxidant gene nuclear factor erythroid 2–related factor 2 (Nrf-2) and undergo proteasomal degradation [38,39]. Cellular stress also increases the expression of the ubiquitin-specific peptidase-10 (USP10) gene. The USP10 gene stabilizes p53, increasing apoptosis of wounded keratocytes. However, it also deubiquitinates integrins, contributing to the activation of TGF-β and fibrosis [40]. The apoptotic cells also release TGF-β and other inflammatory cytokines, completing the loop of fibrosis [41,42] (Figure 2).

The complex signaling cascade signifies the corneal wound healing and scarring process. The corneal scarring process continues even after the wound has healed, making the condition worse. Keratocytes return to their quiescent state after wound healing, and the immune cells disappear. However, the disrupted Bowman’s membrane does not regenerate because of its low regenerative capacity. This causes extracellular vesicles and persisting fibroblast cells to continuously invade the stromal layer, accumulating TGF-β and repeating corneal ECM remodeling. Therefore, a scarred cornea is an immediate outcome of the multiplexed corneal wound-healing process [43].

Good vision requires a healthy corneal stromal ECM. The wound-healing process replaces the clear transparent stromal tissues with fibrotic tissues containing a disorganized collagen fibril arrangement and different proteoglycan content. There is an increase in the synthesis of chondroitin sulfate, hyaluronan, and biglycan and a decrease in the synthesis of keratin sulfate and lumican, which are required for maintaining corneal transparency [44,45]. This disordered, newly remodeled corneal ECM also lacks normal curvature and possesses low mechanical strength and elasticity. It refracts the incident light in all directions; therefore, the light is not focused on the retina, leading to vision impairment [46]. Externally stimulating the body’s response in attenuating the signaling cascade of corneal fibrosis might be a better approach to recovering a scarless cornea.

## 3. Regeneration of Scarless Cornea

Restoring useful vision is the primary concern of patients with corneal scarring. This can be achieved by preventing the excessive migration of corneal ECM components during wound closure. TGF-β, the growth factor responsible for disrupting the corneal ECM, must remain inactive during the wound-healing process. This review focuses on various therapeutic tools, such as exosomes, small oligo-nucleotide sequences, and bioactive molecules that can either directly interrupt the TGF-β signaling pathway, prevent the secretion of active TGF-β, or modulate a secondary molecule that disrupts the TGF-β signaling cascade. Detailed mechanistic knowledge of these therapeutic tools is required to understand their true potential and overcome current limitations. Successfully implementing these techniques might eliminate the need for corneal transplantation.

### 3.1. Exosomes as A Therapeutic Tool

Exosomes are membrane-bound vesicles released by cells into their extracellular space. They have emerged as a novel therapeutic approach over cell-based therapy for treating corneal scarring [47]. Most stem-cell-based therapies face several challenges, such as immunogenicity, the high cost of production, ethical concerns, stability, and survival. However, exosomes have less stringent safety and regulatory requirements than other stem-cell-based therapies. These microvesicles act as biological vehicles that deliver parent cell cargo to the target cells. The exosomes are internalized by the target cells by either receptor-mediated endocytosis or simple membrane fusion. Upon reaching the cytoplasm of the target cell, the exosomes release their contents, activating various signaling cascades [48]. Exosomes can be isolated from stem cells using simple techniques, such as ultracentrifugation, ultrafiltration, size exclusion chromatography, and immunity capture. These vesicles can deliver antifibrotic proteins and miRNAs from stem cells to the ocular surface to modulate the therapeutic signaling cascade of the scarred cornea.

An increase in the concentration of chemokines and cytokines at the injury site is the hallmark of wound healing. Interleukin 8 (IL-8), a proinflammatory cytokine, binds to glycosaminoglycans (GAGs). In this GAG-bound form, IL-8 interacts with the C-X-C motif chemokine receptor 1 (CXCR1) and CXCR2 of neutrophils that infiltrate at the site of injury, leading to oligomerization and a haptotactic gradient. This haptotactic gradient directs the migration of neutrophils to the wounded area and seeds the process of corneal fibrosis [49]. Corneal stromal stem cells (CSSCs) express tumor necrosis factor (TNF)-stimulated gene 6 (TSG-6), a hyaluronan-binding protein, during inflammation [50]. This 35 kDa protein interacts with IL-8 via its link module domain and disrupts the binding of IL-8 and GAGs, preventing neutrophil migration [51,52]. Shojaati et al. [53] isolated extracellular vesicles (EVs) from CSSCs, mixed them with fibrin gel, and administered the EV–fibrin gel to the surface of an eye with corneal debridement. After 2 weeks of debridement, the EV-treated cornea showed minor corneal scarring compared to CSSC-treated and untreated controls. EVs were more effective in preventing neutrophil infiltration, reducing fibrotic marker expression, and restoring corneal morphology.

Cultured CSSCs treated with exosomes isolated from adipose-derived stem cells (ADSCs) showed optimal proliferation, reduced apoptosis, increased aldehyde dehydrogenase 1 (ALDH1), decreased MMP1, MMP3, and MMP9 expression, and increased collagen I, II, III, IV, and V expression compared to untreated CSC [54]. Therefore, ADSC-derived exosomes might revive the plasticity of transformed corneal keratocytes or stromal cells by increasing keratocyte marker expression and ECM production. The therapeutic effects of ADSCs and ADSC-derived exosomes could be attributed to microRNA-19a, which binds the 3′UTR of homeodomain-containing serine/threonine kinase 2 (HIPK2). MicroRNA-19a post-transcriptionally suppresses homeodomain-interacting protein kinase 2 (HIPK2), preventing the activation of the Jun N-terminal kinase (JNK) pathway and fibrosis [55]. Corneal keratocytes, when co-cultured with ADSC-derived exosomes, reduce HIPK2, phosphorylated SMAD3, and p53 expression, preventing the transformation of keratocytes into myofibroblasts and apoptosis of the nearby injured cells. This condition was reversed by the lentivirus-mediated overexpression of HIPK2 in keratocytes, confirming the role of miRNA-19a in corneal scar healing [56].

In a study, human corneal epithelial cells (HCEs) were treated with recombinant thrombospondin 1 (TSP1) to evaluate the effectiveness of corneal epithelial-derived exosomes in corneal wound healing. They were subjected to the same hypoxic stress encountered in corneal wound healing. Exosomes with TSP-1 as a cargo protein decrease paraptosis (apoptosis caused by hypoxic conditions) [57]. EVs derived from human placenta mesenchymal stem cells were used to treat alkali-burned mouse corneas. The EVs decreased vascular endothelial growth factor (VEGF) expression after 48 h, and corneal restoration was observed 14 d post-treatment. The HCEs treated with EVs showed a reduction in the expression of profibrotic genes (IL-10, IL-1β, TNF-α, and NF-κβ) and the apoptosis gene cas8, revealing their anti-inflammatory and anti-apoptotic potential [58]. The therapeutic effect of this exosome is due to the reduction in inflammation and apoptosis. Increased apoptosis in nearby wounded cells attracts inflammatory molecules and neutrophils. These neutrophils are entrapped in fibrous DNA-containing structures and release neutrophil esterase (neutrophil esterase trap), enhancing the transformation of keratocytes into myofibroblasts, mediated by TGF-β [59,60].

The ECM also possesses factors that can reduce proinflammatory cytokines upon receiving an inflammatory insult and attract macrophages, dendritic cells, and lymphocytes to the injured site. Yin et al. [61] processed the ECM from lymph, cartilage, and corneal tissues into micro-sized particles and compared their efficiency in corneal wound healing. The treatment of IL-1β-pretreated rabbit corneal keratocytes with these ECM microparticles reduced proinflammatory cytokine expression and prevented keratocytes from transforming into myofibroblasts. However, the keratocytes were more elongated than native keratocytes and ECM microparticles in the untreated control group. They also addressed the efficacy of ECM microparticles in modulating the gene expression of the tear film or lacrimal gland. Inflammation decreases tear production; increases tear film osmolarity and osmotic stress; and activates MAP kinase and NF-κβ signaling cascades, causing various inflammatory mediators to aggravate the ocular surface damage [62]. The rabbit cornea was injured by performing a superior lamellar keratectomy. Treatment with fibrin gel (FG)-encapsulated lymph node extracellular matrix microparticles increased Mucin 5AC (Muc5AC) and lactoferrin expression, causing infiltration by neutrophils, a reduction in corneal haze, a reduction in TGF-β, α-SMA, and COL-1 expression, and an increase in the thickness of the mucin layer. Therefore, tissue-derived microparticles could be potent therapeutics for ocular surface homeostasis and prevent corneal fibrosis.

**Figure 3:**
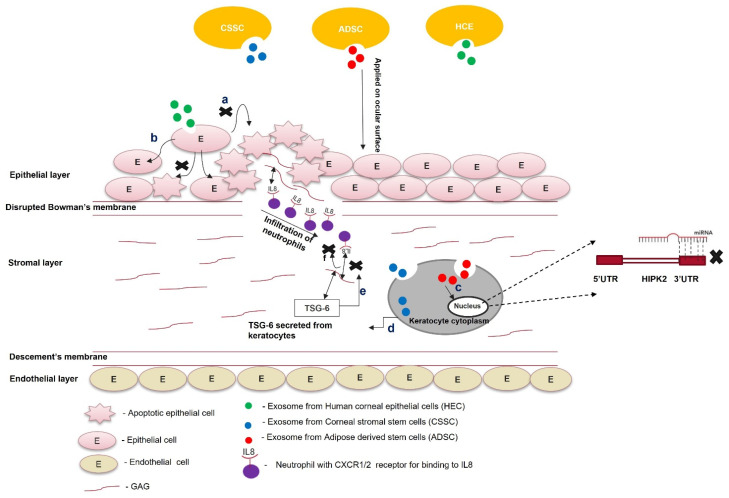
Role of exosomes in the treatment of scars. Exosomes from different cell types (ADSCs, CSSCs, and HECs) are loaded within the fibrin gel or encapsulated in the hydrogel and applied on the ocular surface; (**a**) exosomes from HECs taken up by the epithelial cells prevent paraptosis (apoptosis caused by hypoxic conditions); (**b**) epithelial cells treated with exosomes do not undergo apoptosis; (**c**) exosomes from ADSCs contain miR-19a, which post-transcriptionally silences HIPK2 and attenuates the JNK pathway; (**d**) exosomes from CSSCs contain TSG-6 protein, and once they are taken up by keratocytes, the TSG-6 proteins are secreted within them; (**e**) TSG-6 protein binds to GAG via its LINK domain and prevents the binding of IL8 to the GAG of corneal stroma; (**f**) the unbound form of IL-8 cannot be presented to the CXC1/2 receptor of neutrophils. It attenuates the infiltration of neutrophils to the wounded area of the cornea.

Exosome-based therapy is still in its nascency and requires preclinical research to be used for treating scars. Exosomes isolated from corneal stromal stem cells effectively prevent corneal scarring and require more preclinical safety and toxicity studies. Similarly, ADSC-derived exosomes reduce apoptosis and restore keratocyte marker expression in corneal stromal cells. Further characterization of these experiments is required to check their preclinical efficacy and gain better molecular insight to understand their therapeutic benefits. Exosomes from HCEs can hasten the corneal wound-healing process and prevent corneal scarring (Figure 3). The status of exosome-based therapy for corneal scar treatment is summarized in Table 1. Moreover, the parent cells could be engineered to overexpress specific antifibrotic proteins. Exosomes consisting of this protein can be used in combination with tissue-derived microparticles to increase the effectiveness of exosome-based therapy for a scarred cornea.

### 3.2. Targeted Gene Silencing for the Generation of A Scarless Cornea

The idea of directly targeting the upregulated genes of the corneal scarring signaling cascade to heal a scarred cornea has attracted researchers over the past few years. Semaphorin 3A (SEMA-3A), ubiquitin-specific protease-10 (USP 10), and calmodulin/calcium-activated K+ channel 3.1 (Kca3.1) are upregulated during the corneal wound-healing process. The increased expression of these genes contributes to corneal fibrosis. Therefore, targeting these genes using siRNA can mitigate the scarred corneal environment. The complete knockout of a targeted gene is undesirable because genetically modifying a mammalian cell is difficult, time-consuming, and expensive; moreover, knocking out a gene could have lethal consequences. Therefore, the in vivo delivery of small oligonucleotide sequences, designed to target certain mRNAs, using a suitable delivery vehicle to silence a target gene might be an effective way to prevent corneal fibrosis.

#### 3.2.1. Targeting Semaphorin 3A in Scarless Cornea Regeneration

The epithelial cells around the wounded area secrete various growth factors in response to an injury. Epidermal growth factor-1 (EGF-1) is one such factor that binds to EGF-1 receptors (expressed in large numbers) on the surface of fibroblasts [63]. The binding of EGF-1 to its cognate receptor on the fibroblast of the epithelial layer or the stromal layer of the cornea increases the expression of Semaphorin 3A (SEMA3A). The semaphorin family consists of a large group of proteins that can be either secreted or cell surface-bound [64]. Jeon et al. [65] showed the increased expression of SEMA3A in corneal epithelial and stromal keratocyte cells, indicating its overexpression during wound healing. Similar SEMA3A expression was observed in cultured corneal fibroblasts. Increased expression was observed in fibroblasts pretreated with TGF-β. SEMA3A, in combination with TGF-β, leads to a significant increase in profibrotic marker gene expression, indicating that SEMA3A only supports the TGF-β-mediated expression of fibrotic genes.

Morishige et al. [66] also showed a similar increase in SEMA3A expression in epithelial cells during wound healing. Native epithelial cells were transfected with siRNA targeting SEMA3A; however, its expression was unaltered. In fibroblasts transfected with similar siRNA, there was a reduction in all of the isoforms of SEMA3A, confirming that wound healing increases the level of SEMA3A in fibroblasts. The underlying mechanism behind this role of SEMA3A is still unclear. Yamazaki et al. [67] showed that SM-345431 (vinaxanthone), a SEMA-3A inhibitor, promoted neural regeneration in a murine dry-eye model. This inhibitor also maintained corneal epithelial integrity and nerve density in the dry-eye model, providing a molecular basis for targeting SEMA-3A.

#### 3.2.2. Silencing USP10, A Deubiquitinase, Can Prevent Corneal Scarring

The cellular stress associated with corneal wound healing increases the local levels of USP10, a deubiquitinase with dual function. The elevated USP10 is translocated into the nucleus immediately after the wound. It binds to p53 to prevent cellular ubiquitylation [68] and increases apoptosis of the wounded and surrounding keratocytes or epithelial cells, allowing neutrophil and macrophage infiltration. In the later stages of wound healing, when acute cellular stress is relieved, USP10 accumulates in the cytoplasm and either binds to Ras GTPase-activating protein-binding protein (G3BP), a Ras-GTPase-activating protein-binding protein, or the USP10 modulator, which deubiquitinates integrins [69]. This increases the integrin levels on the cell surface and TGF-β-mediated fibrosis. Lipofectamine-mediated transfection of USP10-siRNA in wounded porcine corneas improved the corneal stromal ECM arrangement. It also decreased fibrotic marker (fibronectin and α-SMA) expression and αVβ1,5 integrin expression on the surface of corneal fibroblasts [70]. Bowmil et al. [71] used a self-deliverable siRNA (siRNA) targeting USP10 conjugated to cys3 and cholesterol to treat wounded rabbit corneas. These siRNAs could penetrate to a depth of 324 µm in the corneal stromal layer within 24 h of transfection and reduce the expression of collagen III and fibrotic markers. They also reduced immune cell infiltration and apoptosis in the cells surrounding the wound. This finding supports that molecular therapeutic tools should target USP10 for scarless corneal wound healing.

#### 3.2.3. Knockout of Kca3.1 Ion Channel for Preventing Corneal Scarring

Ca^2+^ signaling is integral to corneal fibrosis. The axonal nerve endings of the cornea secrete neuropeptides such as substance P (SP) in response to injury. Substance P (SP) binds to NK1Ron on keratocytes and activates phospholipase C-β, which cleaves phospholipid 2,3-bisphosphate to inositol-3-phosphate (IP3) and diacylglycerol. IP3 induces intracellular calcium by opening the IP3-gated Ca^2+^ ion channels of the endoplasmic reticulum. Calmodulin is a Ca^2+^-binding protein that binds to Ca^2+^ ions and activates the Kca3.1 ion channel [72]. The activated K^+^ ion channels cause an efflux of K^+^ ions and an influx of Ca^2+^ ions, resulting in the hyperpolarization of keratocytes. Hyperpolarization activates the transformation of keratocytes into fibroblasts and enables their evasion of the G1/S cell cycle checkpoint, which increases fibroblast proliferation. This increased fibroblast proliferation expedites corneal wound healing, leading to TGF-β-mediated myofibroblast formation, which results in corneal fibrosis [73,74]. Integrin on the cell surface is activated upon binding to specific ligands [75]. It forms macromolecular complexes with ion channels and helps in its localization to the plasma membrane of cells [76]. The increased integrin expression observed on the keratocyte cell membrane during corneal fibrosis indicates an increased accumulation of Kca3.1 on the plasma membrane. The accumulated Kca3.1 hyperpolarizes the keratocytes, contributing to fibrosis. Kca3.1 knockout mice showed reduced haze formation because of reduced fibrotic gene expression and low α-SMA [77]. Human corneal fibroblasts (HCFs) pretreated with TGF-β and incubated with TRAM-34 showed a decrease in profibrotic gene expression compared to only-TGF-β-activated HCFs. Therefore, it is likely that Kca3.1 inhibition prevents the differentiation of keratocytes into myofibroblasts and corneal fibrosis [78]. The targeted silencing of genes such as SEMA3A, USP10, and Kca3.1 can prevent the differentiation of keratocytes into myofibroblasts in the presence of TGF-β, preventing corneal scar formation (Table 2).

### 3.3. Protein Overexpression in Preventing Corneal Scarring

The cornea is an ideal tissue for gene therapy, as the ocular surface can be an excellent platform for the topical application of various viral/non-viral gene delivery vectors. The overexpression of specific genes in corneal epithelial or stromal cells that block the TGF-β signaling cascade can prevent scar formation.

#### 3.3.1. Overexpression of KLF4 in Preventing Scar Formation

Epithelial cells express Krüppel-like factor 4 (KLF4), which coordinates the apical and basal polarity of epithelial cells. It suppresses EMT to maintain the plane of epithelial cell division and epithelial cell homeostasis. Corneal injuries lead to the loss of native epithelial markers, such as E-cadherin 1 (CDH1) and zona occludens, and the transformation of epithelial cells to fibroblast-like cells that secrete TGF-β. Fujimoto et al. [79] reported low KLF4 expression in epithelial cells that migrate towards the wound. Human corneal epithelial cells (HCEs) transfected with siRNA targeting KLF4 showed decreased epithelial markers, increased mesenchymal markers, and increased profibrotic gene expression. The lentiviral-vector-mediated overexpression of KLF4 in HCEs showed the increased expression of corneal epithelial cell markers. However, KLF4 overexpression did not affect mesenchymal gene expression, such as fibronectin 1 and N-cadherin 2. KLF4-overexpressing HCEs and the HCE control were pretreated with TGF-β for 30 min. KLF4-overexpressing HCEs showed 20% less SMAD2/3 phosphorylation compared to the HCE control. However, KLF4-overexpressing HCEs incubated with TGF-β for four hours showed SMAD2/3 phosphorylation levels similar to the TGF-β pretreatment levels. Therefore, KLF4 might contribute to scar prevention due to EMT suppression and a reduction in SMAD2/3 phosphorylation, limiting its nuclear localization and TGF-β-mediated corneal fibrosis.

#### 3.3.2. Overexpression of Id3 for Reviving Cornea without Scars

Human corneal stromal fibroblasts overexpressing inhibitor of differentiation 3 (Id3) in the presence of TGF-β prevent myofibroblast formation. Id3 overexpression also reduces the expression of fibrotic genes, such as fibronectin, α-SMA, collagen I, and collagen IV [80]. After TGF-β treatment, the Id3-overexpressing cells showed: TGF-β binding to its cognate receptors (TGF-βRII and TGF-βRI); the phosphorylation of SMAD2/3 and its translocation to the nucleus; and the activation of transcription factors, such as the ZEB, SNAIL, and basic helix-loop-helix (bHLH) families [81]. The bHLH transcription factor consists of two alpha helixes separated by a loop and a specific DNA-binding domain (DBD). The DBD binds to the E-box DNA consensus sequence and promotes EMT [82]. The Id3 gene, belonging to a class V group of the bHLH family, consists of an HLH domain. However, it lacks the DNA-binding domain and forms dimers with other bHLH transcription factors. The sequestration of the bHLH transcription factor prevents it from binding to the E-Box and thereby suppresses EMT. Therefore, Id3 acts as a dominant-negative regulator of this transcription factor [83]. Id3 overexpression prevents the TGF-β-mediated profibrotic cascade in HCF. However, an in vivo animal-based study is needed to establish its efficacy in reducing corneal scarring.

#### 3.3.3. Overexpression of SMAD7 for Regenerating Cornea without Scars

SMAD7 is a TGF-β signaling pathway inhibitor that binds to TGF-βR1 via its MH2 domain. This binding prevents SMAD3 phosphorylation and its interaction with SMAD2 and SMAD4, inhibiting the nuclear localization of the SMAD complex and TGF-β-mediated fibrosis [84,85]. Gupta S et al. [86] reported an increase in the α-SMA-positive cell count in HCFs transfected with siRNA targeting SMAD after TGF-β treatment. The α-SMA-positive cell count was reduced in TGF-β-treated HCFs after transfection with the recombinant AAV5-SMAD7 viral vector. Rabbit corneal wounds treated with recombinant AVV5-SMAD7 resulted in reduced corneal haze and profibrotic gene expression after four weeks. Moreover, there were no signs of infection, intraocular inflammation, redness, or ocular discharge, establishing the safety and efficacy of this formulation. Therefore, recombinant AVV5-SMAD7-mediated gene delivery is safe and therapeutically efficient in preventing corneal scarring.

An overview of antifibrotic genes with the potential to heal corneal scarring is summarized in Table 2. Further validation of their effectiveness and safety in preclinical and clinical studies is required. Establishing this antifibrotic environment in corneal cells using a gene therapy approach is restricted by the lack of long-term stability, high costs, and low efficiency. Therefore, various bioactive compounds, such as histone deacetylase (HDAC) inhibitors, glucosamine, and chitosan, with the potential to induce similar antifibrotic conditions must be considered.

### 3.4. MicroRNA Therapies in Regenerating Cornea without Scars

MicroRNAs are 21-nucleotide-long, non-coding RNAs that can post-transcriptionally silence the expression of their target genes. These microRNAs form an RNA-induced silencing complex (RISC) with Argonaute proteins. These proteins guide them towards the target mRNA, where they pair with the target mRNA and silence its expression [87].

MicroRNAs in the cornea affect various cellular processes, such as cell migration, differentiation, proliferation, and metabolism. Any dysregulation in miRNA levels during corneal injury can lead to corneal neovascularization and scar formation. An J et al. [88] observed a sharp decrease in the expression of miR-204 in a murine corneal wound model. MicroRNA-204 is abundant in the cornea. It inhibits corneal cell proliferation by G1 phase arrest. Therefore, low miR-204 levels during corneal wounds account for the excessive proliferation of transformed epithelial cells, myofibroblasts, ECM remodeling, and fibrosis during quick wound healing. Wang Y et al. [89] compared lens epithelial cells from healthy donors and patients with posterior capsular opacification (PCO). They showed increased α-SMA and vimentin and decreased E-cadherin in the diseased cells, indicating opacification. An in vitro PCO model was developed and transfected with miR-204. The lens epithelium cells showed increased E-cadherin and decreased α-SMA compared to non-transfected controls and miR-204-5p-inhibitor-transfected cells. MicroRNA-204 prevents TGF-β-mediated EMT and fibrosis by targeting SMAD4, disrupting the SMAD2/3-SMAD4 complex. It also decreases the expression of the Hey/HMGA transcription factors, which induce EMT through CDH1 repression and SNAIL activation [90,91]. A subconjunctival injection of a recombinant adeno-associated vector (rAAV-miR-204) prevented neovascularization in murine alkali-burned corneas, indicating the anti-angiogenic role of miR-204 [92].

Ratuszny et al. [93] showed the significant upregulation of miR-145- and TGF-β-induced myofibroblasts in scarred human corneas compared to normal corneal and untreated corneal fibroblasts. The TGF-β-mediated upregulation of miR-145 in the myofibroblast induces α-SMA expression by downregulating KLF4. miR-145 mediates the post-transcriptional silencing of KLF4 by targeting its 3’UTR [94]. miR-145 silencing using anti-miR-145 resulted in decreased α-SMA and increased KLF4 expression. The lipofectamine-mediated transfection of a miR-145 inhibitor caused the stimulated cells to reduce collagen gel contraction in TGF-β-pretreated corneal fibroblast cells. This miR-145 inhibitor decreased the migratory capacity of myofibroblasts by 50%. Sun et al. [95] reported that miR-145 can directly target the 3′UTR of KLF4, mediating post-transcriptional gene silencing. KLF4 is an EMT suppressor that interacts directly with the promoter of SMAD7 and upregulates its expression. The increased expression of SMAD7 prevents SMAD2/3 phosphorylation and localization, disrupting the TGF-β signaling pathway [96]. Therefore, miR-145 is a promising target for further therapeutic exploration to prevent corneal scar formation.

Micro-RNA133b modulates connective tissue-like growth factor (CTGF), a downstream molecule of the TGF-β signaling pathway [97]. CTGF is a member of the CNN family of proteins. It has an N-terminal insulin-like growth factor-binding protein domain that binds to IGF receptors, accelerating cellular differentiation and ECM secretion. Their C-terminal domain increases cellular proliferation and DNA synthesis in the presence of epidermal growth factor (EGF) and its receptor [98]. Zhao et al. [99] showed that miR-133B conjugated to gold nanoparticles (AuNPs) reduced the expression of myofibroblast-inducing genes (α-SMA and type 1 collagen) in corneal stromal cells. The transplantation of miR-133B/AuNPs with collagen in the rabbit cornea after lamellar keratoplasty inhibited corneal haze and scarring.

**Figure 4:**
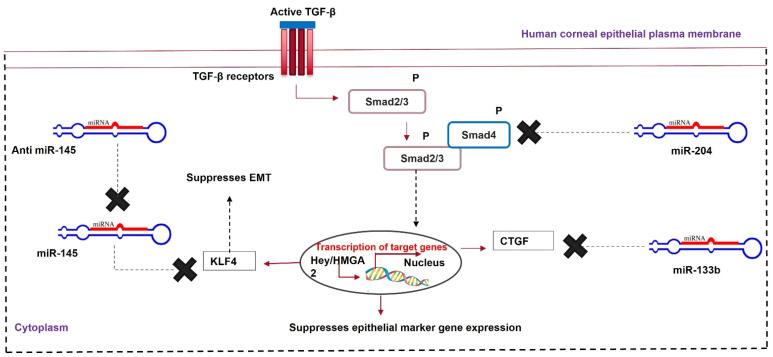
MicroRNA therapy for corneal scarring. Upregulation of miR-204 and miR-133b and downregulation of miR-145 help in healing a scarred cornea via modulating Krüppel-like factor 4 (KLF4), Smad2/3, and CTGF.

The downregulation of miR-145 and the upregulation of miR-204 and miR-133b can prevent TGF-β-mediated corneal fibrosis (Table 3). MicroRNA-based gene targeting is a promising therapeutic approach to prevent and heal corneal scarring (Figure 4).

### 3.5. Bioactive Molecules as HDAC Inhibitors in the Regeneration of Scarless Cornea

The wound-healing process of an injured cornea involves three overlapping phases: the inflammatory phase, the proliferative or fibrotic phase, and the final remodeling phase [100]. These events change the native morphology of the corneal stromal ECM, rendering it opaque. During the initial inflammatory phase of the healing process, corneal cells exhibit high levels of proinflammatory cytokines. During the initial phase of wound healing, the infiltrated neutrophils, monocytes, or activated macrophages secrete TGF-β, increasing its local concentration around the wounded area. TGF-β guides the transition of the wound from the inflammatory phase to the fibrotic phase by decreasing the local proinflammatory cytokine level. The timely termination of the initial inflammatory phase is required for the cells to enter the cell proliferation and ECM synthesis phase, healing the wound [101]. A lesser-known pathway that TGF-β uses for this transition is by modulating histone acetylation. TGF-β recruits histone deacetylases (HDACs), removing the acetyl group from the lysine residues of histones H3 and H4 of anti-inflammatory genes. Histone acetylation is associated with anti-inflammatory gene activation; therefore, HDAC is recruited in the presence of TGF-β, deactivating various anti-inflammatory genes and enabling the transition to the fibrotic phase [102,103,104,105,106]. Trichostatin A (TSA), a deacetylase inhibitor, prevents the deacetylation of histone H3 or H4 of anti-inflammatory genes. TSA prevented the TGF-β-mediated transformation of corneal fibroblasts to myofibroblasts in vitro and also reduced the corneal opacity in a rabbit model of corneal haze [107,108].

A possible mechanism of action for HDAC inhibitors is increasing the local concentration of TGF-β during wounding, leading to HDAC recruitment, SMAD7 deacetylation, SMAD7 ubiquitinylation, and proteasomal degradation. This paves the way for TGF-β-mediated corneal scarring. Similarly, suberoylanilide hydroxamic acid (SAHA), an HDAC inhibitor, reduced α-SMA and MMP9 expression in equine corneal fibroblasts treated with TGF-β. This shows that SAHA can prevent TGF-β-mediated corneal fibrosis [109]. Epigenetic modulators, such as TSA and SAHA, might be useful bioactive molecules for treating corneal scars (Table 4).

### 3.6. Guided Wound Healing to Prevent Scarring

**Figure 5:**
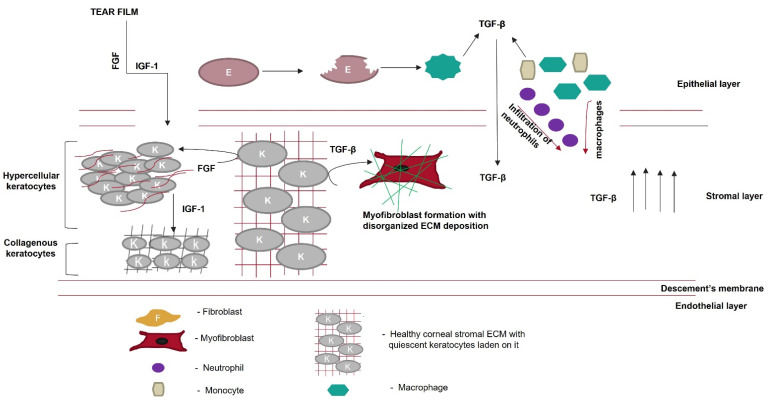
Role of growth factors in scar prevention. Various growth factors, such as fibroblast growth factor (FGF) or insulin-like growth factor (IGF-1), are secreted from the tear film. These growth factors cross the disrupted Bowman’s membrane to reach the corneal stromal layer. FGF converts keratocytes to hypercellular keratocytes. IGF-1 converts hypercellular keratocytes to collagenous keratocytes, which secrete ECM components similarly to the native cornea. Meanwhile, in the epithelial layer, after wounding, epithelial cells undergo apoptosis, and the apoptotic epithelial cells release TGF-β. Neutrophils, macrophages, and monocytes, which migrate from the epithelial layer to the stromal layer, secrete TGF-β, thereby increasing its local concentration. Keratocytes in the presence of TGF-β are converted to myofibroblasts, which disrupt the highly organized fibrillar arrangement of the corneal ECM.

Corneal wound healing is accompanied by the secretion of various growth factors. During wound healing, these growth factors interact with their cognate receptors present on the surface of the cell to regulate the synthesis of collagen, proteoglycan, and other ECM components’ deposition. Various growth factors, such as IGF-1, platelet-derived growth factor (PDGF), TGF-β, and fibroblast growth factor 1 (FGF-1), either reach the wound site from the tear film or are secreted by the surrounding wounded or apoptotic epithelial cells. Etheredge et al. [110] reported that FGF-1 stimulates keratocyte proliferation; however, it inhibits the synthesis of type 1 procollagen and keratan sulfate. Type 1 procollagen is an essential component of the stromal ECM, and keratan sulfate directs collagen assembly to maintain corneal transparency. FGF-1 induces the formation of hypercellular keratocytes with densely packed cells in the sparse matrix. IGF-1 turns these hypercellular keratocytes into collagenous keratocytes that secrete ECM components, similar to native keratocytes. Therefore, the IGF-1/IGF-receptor signaling pathway prevents the transformation of keratocytes into myofibroblasts and ECM remodeling (Figure 5). However, this is not a predominant signaling pathway during wound healing because the local concentration of TGF-β in the wound microenvironment is high. Therefore, the TGF-β/TGF-βRI pathway takes charge, and TGF-β binds to its receptor on keratocytes to synthesize biglycan, fibronectin containing extra domain A, and hyaluronan. This proteoglycan disrupts the spacing between collagen fibrils, reducing the corneal transparency. It also guides the differentiation of keratocytes into myofibroblasts, characterized by the neo-expression of α-SMA. α-SMA incorporates actin into collagen fibrils and exerts a contractile force on the ECM, disrupting the normal corneal architecture.

Therefore, an increase in the local concentration of IGF-1 during corneal wounding can provide insights into this pathway because this pathway directs the synthesis of fibrillar collagen similarly to the native one. This process of guided wound healing avoids scar formation (Figure 4). Sarenac et al. [111] reported that keratocytes treated with TGF-β and IGF1 showed significantly lower SMAD3 nuclear localization and higher SMAD7 compared to TGF-β-treated keratocytes. IGF-1 also increased keratocyte markers, such as keratocan or ALDH3, when TGF-β-pretreated keratocytes were incubated with IGF-1 alone or together with SAHA. The TGF-β-pretreated keratocytes incubated with IGF-1 and halofuginone showed high proliferation and no myofibroblast trans-differentiation. Ghiasi et al. [112] used hyaluronic acid as a vehicle to deliver substance P and IGF-1 to the surface of a photo-ablated rabbit cornea. IGF-1 and substance P synergistically promoted epithelial wound healing after a week of treatment, with a marked decrease in the wounded areas.

TGF-β heals corneal wounds and is a hallmark of the process of scarring. In contrast, IGF-1 guides the process of wound healing and circumvents scarring (Table 4). So far, IGF-1 has been studied as an external growth factor, either alone or together with a bioactive compound, such as SAHA or substance P. Bioengineering keratocytes to overexpress IGF-1 can increase its local concentration and allow it to control the process of wound healing.

### 3.7. Clinical Therapy for Scar Prevention

A gold-standard technique to prevent corneal scarring is still under research. Various biomolecules, such as decorin, TPCA-1, glucosamine, acetylcholine, and chitosan, have the potential to heal a scarred cornea via their respective biological pathways. Chen et al. [113] reported the significance of glucosamine in corneal scar treatment. This amino sugar finds clinical application in osteoarthritis management [114]. However, it significantly enhances intraocular pressure [115]. Park et al. [116] noted that glucosamine attenuated renal fibrosis by downregulating SMAD2 phosphorylation; therefore, a study of glucosamine’s effects on corneal fibrosis can provide new insights into its clinical applications. Chen’s group observed an increase in the expression, stability, and nuclear localization of KLF4 and a decrease in fibrotic gene expression in HCFs treated with glucosamine and TGF-β. The mechanism underlying the glucosamine-mediated increase in KLF4 expression is still under research. However, a study by Wang DF et al. [117] reported that glucosamine increases the expression of the phosphorylated form of PTEN (a phosphatase), leading to an increase in the phosphorylated form of KLF4. This increases the P300 histone acetylase activity, mediating H3 histone acetylation [118,119].

**Figure 6:**
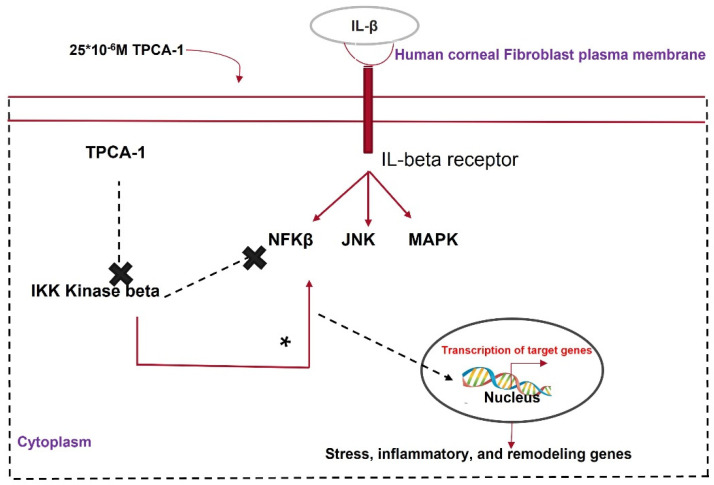
Mechanism of action of TPCA1 in attenuating corneal fibrosis.

Glucosamine can form O-linked N-acetylglucosamine via glucosamine transferase activity and the post-translational modification of the proteasome complex to prevent KLF4 degradation [120]. Moreover, KLF4 interacts with TGF-β control elements and prevents a proinflammatory environment [121]. Therefore, glucosamine might find clinical applications in healing corneal scarring (Figure 6).

Chitosan is an effective therapeutic tool for wound healing because of its antifibrotic and anti-angiogenic properties and its ability to promote rapid re-epithelization for wound closure [122,123]. Fischak et al. [124] formulated a topical eye drop for corneal epithelial wound dressing by functionalizing chitosan with N-acetylcysteine to improve its mucoadhesive properties. They observed faster wound closure in a rabbit corneal wound model. Similarly, thiolated chitosan nanoparticles (TCS-NPs) prevented myofibroblast formation in IL-6-treated HCFs [125]. The TCS-NPs reduced fibronectin or collagen I expression and VEGF expression by 99.9%. Chitosan downregulated TGF-β expression by promoting a more stable interaction between miRNA-29b and the CDS region of TGF-β, attenuating TGF-β signaling [126]. However, the expression of miRNA-29b was not upregulated after TCS-NP treatment. The cellular pathway underlying the chitosan-mediated attenuation of TGF-β expression remains unclear; however, functionalized chitosan can be a novel therapeutic approach to prevent corneal scarring.

Lycium barbarum polysaccharide (LBP), derived from the ancient herb *Lycium Barbarum*, is a bioactive molecule clinically used in kidney, eye, and liver fibrosis [127]. Du et al. [128] observed that LBP prevented UV-B-induced apoptosis of corneal epithelial cells (CECs) by preventing the phosphorylation and activation of the JNK pathway. In a proof-of-concept experiment to test LBP’s efficacy in attenuating corneal fibrosis, TGF-β stimulated HCF cells subjected to varying concentrations of LBP showed a dose-dependent decrease in α-SMA expression and a reduction in profibrotic collagen [129].

Fibrosis is generally accompanied by the production of reactive oxygen or nitrogen species (ROS/RNS) [130]. ROS are produced because of the TGF-β-mediated activation of NADPH-oxidase 4 via the SMAD3 signaling pathway [131]. ROS directly contribute to fibrosis by inducing TGF-β expression [132]. They also indirectly contribute to fibrosis by activating various growth factors, chemokines, cytokines, and other inflammatory molecules [133]. Increased ROS levels have been observed in skin specimens of patients with systemic sclerosis or skin fibrosis [134]. This increase is associated with EMT and myofibroblast accumulation [135]. Therefore, attenuating ROS production might prevent scarring events. Nuclear factor erythroid 2–related factor 2 (Nrf2), a leucine zipper transcription factor, decreases ROS production by increasing the expression of antioxidant genes, such as superoxide dismutase and NADPH quinone oxidoreductase, by binding to the antioxidant response element. Nrf2 is subjected to proteasomal degradation in the presence of kelch-like ECH-associated protein 1 (keap1), which is activated by BRD4 expression triggered by TGF-β. JQ1, an inhibitor of BRD4, reduces the expression of α-SMA and collagen I. It increased Nrf2 nuclear localization in HCF cells stimulated with TGF-β in vitro. It also increased corneal scarring in an injured mouse model by reducing fibrotic marker expression, supporting the antifibrotic role of JQ1 [136]. A similar antifibrotic effect was observed when immortalized HCFs pretreated with TGF-β were transfected with siRNA targeting BRD4, indicating that BRD4 inhibition prevents TGF-β-mediated corneal fibrosis.

IL-β, a proinflammatory cytokine, crosses the disrupted Bowman’s membrane and binds to receptors on keratocytes upon injury, promoting apoptosis or angiogenesis in the cornea [137]. IL-1β activates the NF-κβ pathway, which causes inflammation and fibrosis. The NF-κβ pathway is activated by the phosphorylation of either the IKKα or IKKβ subunit of its key modulator, IKK. TPCA1 inhibits the phosphorylation of the IKKβ subunit and deactivates the NF-κβ pathway. Zhang et al. [138] showed that TPCA1 preserved the keratocyte phenotype in HCFs pretreated with IL-β via nuclear accumulation of phosphorylated NF-κβ. Corneal keratocytes stimulated with IL-1β showed the reduced expression of keratocyte markers such as KERA, LUM, and ALDH3A1 and elevated levels of phosphorylated NF-κβ [139]. IL-1β treatment elevated MMP2, MMP3, and MMP9 expression in corneal fibroblasts. This was reversed by TPCA-1 application to IL-1β-stimulated fibroblasts [140]. MMPs have multiple roles in wound healing, such as ECM remodeling, TGF-β cleavage, and the activation of EMT [141]. TPCA-1 can prevent corneal scarring by inhibiting IKK and MMP (Figure 6).

Acetylcholine is a neurotransmitter that is released by cholinergic receptors. CECs show a very high expression of acetylcholine [142]. Acetylcholine interacts with its nicotine receptor, causing the influx of Na^+^ or Ca^2+^ ions and the efflux of K^+^ ions. This modulates various signaling pathways through protein kinases and phosphatases [143]. Acetylcholine can also act via a murine receptor (mAchR) to activate multiple downstream signaling pathways by activating protein kinase C. mAchR (M1, M3) and nAchR are present in CECs, and acetylcholine can promote re-epithelization in an injured rat cornea [144]. In contrast, keratocytes produce a meager amount of acetylcholine. Acetylcholine promotes keratocyte proliferation by activating the mAchR present on keratocytes. Sloniecka et al. [145] showed reduced fibrotic gene expression in primary human keratocytes cultured in the presence of acetylcholine with concentrations of 10^−7^ M and 10^−8^ M. The low dose of acetylcholine had a pronounced antifibrotic effect, indicating that it can be developed as topical eye drops for safe and antifibrotic wound healing.

Decorin, a prime proteoglycan of the corneal ECM, can interact with all three isoforms of TGF-β. It can directly sequester the active form of the growth factor and attenuate its function [146]. The overexpression of decorin using a mammalian expression vector prevents myofibroblast transformation in TGF-β-stimulated HCFs [147]. Decorin binds to its cognate receptor and increases the intracellular calcium level. This increase in calcium activates calmodulin, which phosphorylates CAM Kinase II and inhibits the TGF-β-mediated activation of fibrotic genes via SMAD 2 phosphorylation [148]. The administration of decorin, using fluid gel with a better drug retention capacity, promoted rapid re-epithelization in ex vivo injured rat corneas [149]. Therefore, the ocular administration of decorin using a delivery vehicle with increased retention time exerts a better therapeutic response in regaining a scarless cornea [150]. Table 4 summarizes all of the biomolecules that can be used as therapeutic tools in reviving a cornea without scars.

### 3.8. Nanomedicine in Corneal Scarring Treatment

**Figure 7:**
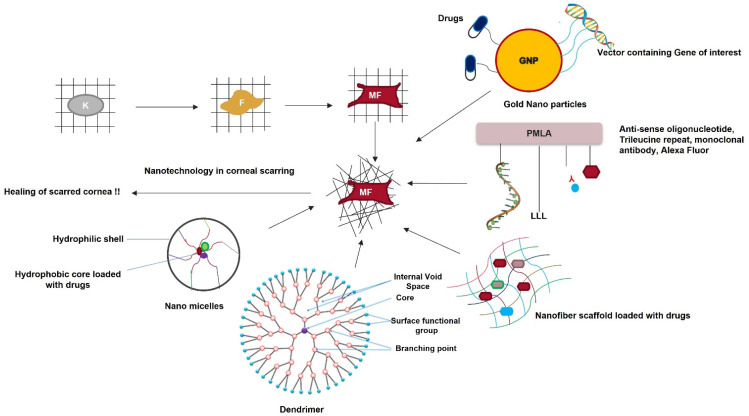
Nanotechnology as a therapeutic tool for reviving scarred corneas. Keratocytes transform into fibroblasts and then into myofibroblasts in the presence of TGF-β. This disrupts the highly organized fibrillar arrangement of the corneal stromal ECM. Nanoparticles loaded with the gene of interest or specific drugs, nanofiber scaffolds loaded with drugs, dendrimers, nanomicelles, and nanopolymers are a few nanomedical approaches for treating a scarred cornea.

Nanomedicine is an emerging field in medical science with the potential to revolutionize corneal fibrosis therapy (Figure 7). Nanomedicine utilizes materials with nanometer dimensions to target living cells at the genetic and molecular levels. It ensures efficient and sustained drug release, increasing the bioavailability of drugs. This is especially good for topical eye drops, which have a bioavailability of only 5% [152].

Ultrafine nano-scale particles or nanoparticles can either be metallic, polymeric, or a conjugate of both. Gold or silver nanoparticles (AuNPs/AgNPs) loaded with genes of interest are excellent biocarriers for gene delivery because of their inert and non-toxic nature in cellular systems [153]. Administering AuNPs loaded with the bone morphogenetic protein 7 (BMP7) gene on the wounded cornea decreased profibrotic gene expression and reduced corneal scarring [154]. Polymers, such as chitosan, polyethylene glycol, albumin, and polyethyleneimine (PEI), are commonly used as carriers in the preparation of nanomedicine. Bhatta et al. [155] showed the sustained release of natamycin on the corneal epithelium surface using chitosan NPs. Sharma et al. [156] prepared hybrid nanoparticles consisting of polyethyleneimine and gold (PEI-AuNP) and loaded them with a plasmid containing BMP7 and HGF genes. These hybrid NPs were administered to alkali-burned corneas. They restored corneal re-epithelization, prevented stromal scarring, decreased profibrotic gene expression, and promoted myofibroblast apoptosis. The hybrid NPs were non-toxic, restored intraocular pressure and tear production, and prevented immune cell infiltration. Similarly, HCFs showed reduced α-SMA and collagen expression after transfection with PEI loaded with siRNA targeting TGF-βRI [157,158]. A liquid crystal nanoparticle system loaded with 0.5% pirfenidone promoted re-epithelization in wounded rabbit corneas without toxicity or irritation [159].

A polymeric acid nanofiber scaffold covalently attached to antisense oligonucleotides targeting diabetes-linked genes was conjugated to a monoclonal antibody for cell targeting. A trileucine repeat for endosomal rupture (caused by low pH) and an Alexa Fluor for cell tracking showed the release of the antisense oligonucleotides in the cytoplasm of the target cell. The oligonucleotides were released because of covalent bond cleavage by cellular glutathione, resulting in the binding and suppression of diabetes-linked genes [160]. Ma et al. [161] prepared a poly-lactic co-glycolic acid (PLGA) scaffold that served as an ideal platform for corneal keratocyte growth and differentiation. This scaffold repaired corneal stromal defects. Polylactic acid nanofiber scaffolds can be loaded with drugs such as cyclosporine A to accelerate corneal stromal wound healing [162].

Dendrimers are branched three-dimensional nanostructures. They can be coupled with polypropylene and crosslinked with collagen. They have high mechanical strength and can serve as an optimal tissue-engineering scaffold for corneal cells [163]. Nanodevice soft lenses can be loaded with suitable drugs for sustained release over time and can be placed on the ocular surface. Pullulan and poly-caprolactone can be used to prepare nanospheres that can be loaded with antimicrobial drugs, such as ciprofloxacin. These nanospheres can be coated on the surface of the lens to prevent *Staphylococcus aureus* and *Pseudomonas aeruginosa* infections in the eye [164]. Nanosponges are highly branched colloidal nanostructures that can be synthesized by crosslinking β-cyclodextrin and di-phenyl carbonate. When loaded with dexamethasone, these nanosponges increase the drugs’ permeability on the ocular surface [165].

Although nanomedicine has proved to be an effective tool for corneal wound treatment, high production costs, tissue accumulation, and in vivo stability are the major limitations of their therapeutic application [166]. Nanomedicine is a promising approach because of its drug release kinetics over conventional drug delivery methods. Therefore, it should be explored further for treating corneal scars.

## 4. Conclusions and Future Perspectives

Corneal wound closure after a major ocular trauma initiates the process of corneal fibrosis, altering a healthy cornea into a scarred one. A scarred cornea is generally characterized by a disorganized opaque stromal ECM laden with myofibroblasts. This causes permanent blindness and affects millions globally each year. Medical practitioners mostly rely on topical ophthalmic drugs (mitomycin C, corticoids, etc.) to treat scarred corneas. These drugs work by either reducing inflammation or decreasing the synthesis of proinflammatory cytokines. However, their low retention time on the ocular surface limits their efficacy. Therefore, there is a need for a more efficient therapeutic approach to healing a scarred cornea.

The explicit targeting of a downstream signaling molecule of the corneal scarring cascade is a contemporary approach to treating the condition. Most of these molecular therapeutics are either in nascent preclinical trials or need further in vivo validation. In exosome-based therapeutic approaches, exosomes isolated from a particular parent cell type hold the potential to transfer the parent cell cargo, such as TSG-6 miRNA (from corneal stromal stem cells), TSP1 (human corneal epithelial cells), or miR-19a (adipose-derived stem cells), to the target cell (wounded keratocytes). The exosomes prevent its conversion to a myofibroblast by either preventing neutrophil infiltration, reducing apoptosis, or blocking the TGF-β signaling cascade.

Most exosome experiments are performed at the cell culture level and possess no clinical validation. The main drawback of this therapy is the proper delivery of the isolated exosomes to the ocular surface, as macrophages engulf most of the exosomes administered to the target organ. Exosomes are loaded in fibrin gel, which adheres to the ocular surface and delivers exosomes. However, exosomes can be encapsulated within hydrogel for more efficient delivery to the target organ [167]. The other limitations of this approach are the large-scale production, isolation, and storage of the exosomes, which need further consideration.

Viral vectors such as lentiviral or adeno-associated viral vectors are commonly used as carriers to deliver targeted therapeutic genes (gene therapy) to the ocular surface. The overexpression of genes such as KLF4 and Id3 suppresses EMT by preventing the phosphorylation of SMAD2/3 and sequestering bHLH transcription factors. Therefore, they prevent the conversion of epithelial cells into fibroblasts, which are further converted into myofibroblasts in the presence of TGF-β. In contrast, the overexpression of SMAD7 blocks the TGF-β signaling cascade by preventing the phosphorylation of SMAD3, averting SMAD2/3 complex formation, and halting fibrosis. Histone deacetylase inhibitors such as SAHA or halofuginone prevent the removal of acetyl groups from SMAD7, stabilizing it to implement its antifibrotic role. These therapeutics prevent the fibrotic healing of the wounded cornea by directly or indirectly blocking the TGF-β signaling pathway.

Another approach to dealing with fibrosis is by diverting the pathway from fibrotic-type healing to a more sophisticated mechanism, which is a guided wound-healing process. This method can be implemented by IGF-1 and its cognate receptor. TGF-β converts keratocytes into myofibroblasts; however, IGF-1/IGF-IR converts keratocytes into collagenous keratocytes. The deposition of native ECM components occurs during the ECM remodeling phase of the wound-healing process.

Small oligonucleotide sequences, such as miRNAs or siRNAs, are used to silence certain targeted profibrotic genes to halt the fibrotic pathway. Semaphorin 3A silencing prevents TGF-β-mediated fibrosis. Similarly, silencing the USP10 gene increases the chances of integrin ubiquitination by cellular ubiquitin. The decreased integrin levels indicate lower chances of activating TGF-β from its latency, halting fibrosis. Clinically accepted blockers of certain ion channels, such as the KCa3.1 ion channel by TRAM34, prevent the hyperpolarization of keratocytes by preventing the influx of Ca^2+^ ions and the efflux of K^+^ ions. This prevents their conversion into myofibroblasts.

The microRNA level of the cornea is disturbed during corneal wound healing. Certain fibrotic miRNAs, such as miR-145, are upregulated, and others, such as miR-205 or miR-133b, are downregulated, creating a scarred corneal environment. Modulating these miRNA levels during fibrotic wound healing can suppress fibrotic gene expression, prevent EMT, and stall fibrosis. Clinically recognized biomolecules are commonly used to treat non-ocular diseases, such as glucosamine, chitosan, acetylcholine, TPCA-1, and decorin. They also hold great potential in treating corneal scarring. Glucosamine, chitosan, and acetylcholine possess inherent antifibrotic activity and attenuate corneal scarring. TPCA1 halts the NF-κβ pathway and prevents corneal fibrosis. The ocular drug decorin phosphorylates the inhibitory serine residues of SMAD2 and forms the inhibitory complex of SMAD2/3/4, which cannot translocate into the nucleus to express fibrotic genes.

Researchers have also explored the emerging field of nanotechnology to develop nanofiber scaffolds, nanoparticles, or nanospheres. These particles are loaded with suitable ocular drugs for increased bioavailability to the ocular surface. Many of these scar dissolution therapies are at the clinical trial stage in various research hospitals.

**Figure 8:**
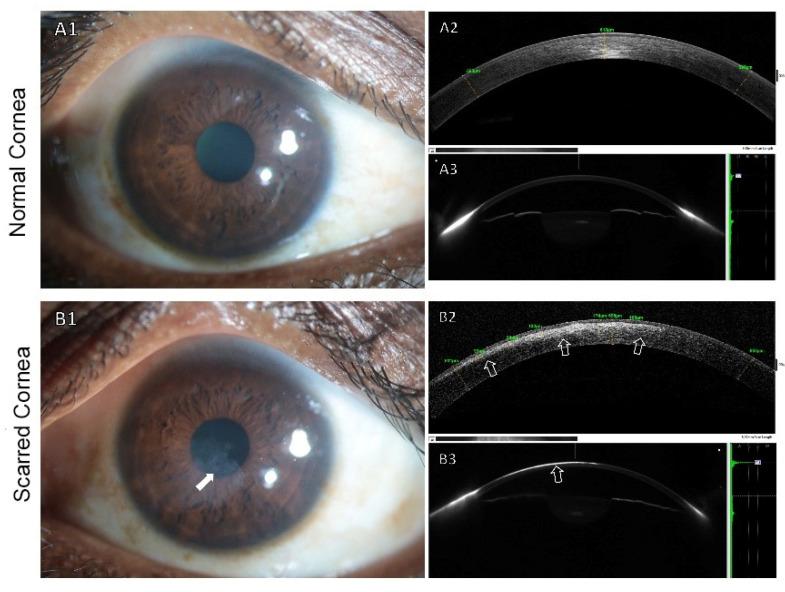
Clinical images of normal and scarred corneas (LVPEI Eye Hospital, Hyderabad, Telangana, India). Clinical appearance of a typical clear cornea and corneal scarring. Slit-lamp photography image (**A1**), optical coherence tomography (OCT) image (**A2**), and Scheimpflug image (**A3**) of the right eye of a patient with a clear cornea. The corresponding images of the left eye with corneal scarring show opacification and haziness on slit-lamp biomicroscopy (white arrow, **B1**). Increased reflectivity in the anterior stroma with thinning and irregularity on OCT imaging (white outlined arrows, **B2**). Increased light scattering on Scheimpflug scanning (white outlined arrow, **B3**).

Figure 8 presents the clinical appearance of the scarred cornea of a patient and how it can be objectively assessed using in vivo imaging. A clinical trial (NCT04932629) using limbus-derived stromal/mesenchymal stem cells to modulate corneal scarring is ongoing in India. For corneal scarring, molecular therapeutics are still in their nascency. A clearer understanding of their effectiveness is required for their clinical translation and therapeutic application.

**Figure 9:**
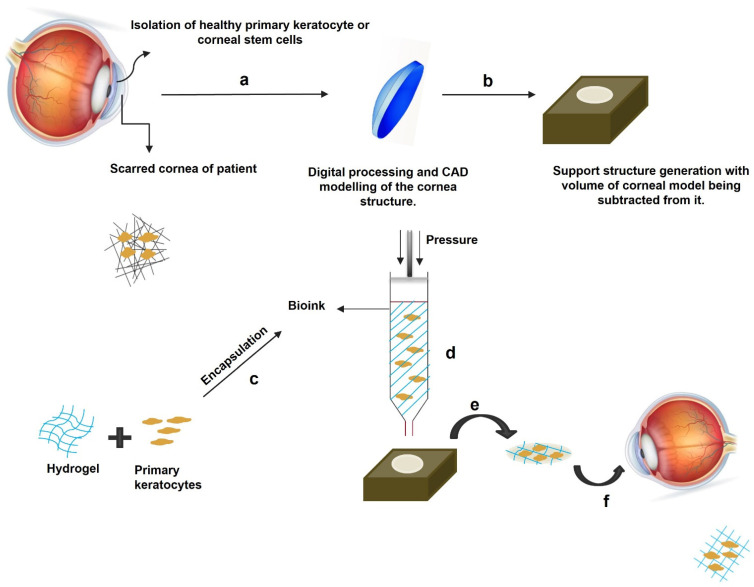
The 3D bioprinting of corneal stroma. The patient with corneal scarring has a disorganized opaque corneal ECM that must be replaced with a 3D-bioprinted corneal stromal layer. The isolation of corneal stromal stem cells or healthy keratocytes from the patient cornea; (**a**) digital image processing of the patient’s cornea to obtain its 3D model, with dimensions that are identical to those of the patient’s cornea; (**b**) generation of a support structure and subsequent substructions of the corneal model volume to obtain a hollow surface mimicking the patient’s corneal structure, onto which 3D bioprinting is carried out; (**c**) preparation of collagen/alginate/gelatin/dECM hydrogel and culturing of either corneal stromal cells or primary keratocytes in appropriate culture media, followed by encapsulation of corneal cells into the hydrogel to prepare the bio-ink; (**d**) loading the bio-ink into the extrusion 3D bioprinter. (**e**) bioprinting the corneal stromal construct. (**f**) transplanted bioprinted cornea in patient’s eyes.

The future lies in the hands of 3D bioprinting. The gap between cornea donors and clinical needs is too wide. Biocompatible 3D-printed corneas can be used for transplantations. This is the most non-controversial method for treating corneal scarring. Collagen, gelatin, alginate, decellularized extracellular matrix, or a combination of these materials can be used as bio-ink to 3D print corneal equivalents. Isaacson et al. [168] created a CAD model of a plastic support structure based on the actual dimensions of the native cornea and coated the inner surface of the support with gelatin microparticles (freeform reversible embedding of suspended hydrogels). These microparticles acted as sacrificial supports for printing the less viscous bio-ink on the CAD model. An extrusion-based bioprinter with a nozzle diameter of 200 µm was used to bioprint the corneal stromal equivalent on the CAD model using a methacrylate-collagen–alginate hybrid bio-ink. The extrusion bioprinter can also be loaded with keratocyte-encapsulated decellularized ECM (dECM) hydrogel. This bio-ink is extruded from a 25G nozzle of the bioprinter. It experiences the shear force at the nozzle tip to spatially align as the corneal equivalent [169]. The strategy can be used to print an aligned matrix that will support better cell growth and corneal ECM remodeling (Figure 9). The 3D bioprinting technique holds the potential to develop a clinically acceptable corneal equivalent that can replace a scarred cornea [170]. However, this warrants the development of a bio-ink with mechanical properties and optical transparency closely mimicking the native cornea. Recreating the highly ordered fibrillar arrangement of the native corneal ECM will undoubtedly be a great breakthrough in corneal research.

## Figures and Tables

**Table 1:** Exosomes as therapeutic tools in reviving a cornea without scarring.

Therapeutic Method	Therapeutic Strategy			Advantages	Limitation	References
Exosomes	Origin	Target Cell	Mechanism of Action	Quick delivery of therapeutic cargo to the target cell. No negative side effects. In the short term, it does not initiate any inflammatory response. In the long term, it directs the cells to modulate themselves to retain the antifibrotic environment.	Quick, large-scale isolation of exosomes from parent cells. Collection of exosomes with high purity. Lack of proper delivery methods to the target cells. Clearance of exosomes inside the cellular environment by macrophages.	[51,52,53,54,55,56,57,58,59]
	Corneal stromal stem cells	Human corneal stromal cells (keratocytes)	TSG-6 protein in the exosomes prevents neutrophil infiltration, reducing the excessive secretion of TGF-β at the wounded area of the cornea			
	Adipose-derived stem cells	Human corneal stromal cells (keratocytes)	miR-19a microRNA in the exosome post-transcriptionally silences HIPK2, halting the JNK fibrotic and TGF-β pathways			
	Human corneal epithelial cells	Corneal epithelial cells	TSP1 protein in the exosomes attenuates paraptosis and helps in wound healing			
Tissue-derived microparticles	Lymph node ECM	Keratocytes	Increases the expression of mucin and lacrimal gland genes (maintains tear film homeostasis), reduces profibrotic gene expression, and reduces corneal haze	Tissue-derived particles can be processed in various physical forms, such as sheets, spheres, and gels, based on clinical needs.	These microparticles are ineffective in epithelial cells under stress and inflammatory environments. Low retention time on the ocular surface.	[60,61,62]

**Table 2:** Targeted gene knockdown and protein overexpression as therapeutic tools for preventing corneal scarring.

Therapeutic Method	Therapeutic Strategy			Advantages	Limitations	References
Targeted gene silencing	Gene Targeted	Mechanism of Action on Targeted Gene	Method of Silencing	Excludes the use of drugs and their associated side effects. Targeted long-term post-transcriptionally silencing of genes without complete knockdown of the gene.	No proper delivery vehicle of siRNA inside the targeted cell because of its negative charge and water solubility, resulting in a poor penetration capacity. Instability of siRNA inside the cellular environment. Digestion of the siRNA by nucleases present in the cell cytoplasm.	[64,65,66,67,68,69,70,71]
	Semaphorin 3A	Potentiates TGF-β to enhance its fibrotic activity	siRNA targeting SEMA-3A			
	USP10	Increases apoptosis by stabilizing p53 (initial stage of wound-healing process) and prevents ubiquitination of integrins by binding to its modulator (G3BP)	siRNA targeting USP10			
	KCa3.1 ion channel	Keratocyte hyperpolarization, resulting in their escape from the G1 phase of cell cycle. This leads to excessive cell proliferation.	TRAM 34, ion channel blocker			
Targeted gene overexpression	Gene Targeted	Mechanism of Action on Targeted Gene	Method of Overexpression	This strategy can be applied to modify both wild and mutant cell types. Non-drug method for treating scarring.	Extra stress on the cell, as cellular resources are wasted in translating and exporting the specific protein. Instability of plasmid DNA expression vectors containing the gene of interest or misincorporation of the gene of interest in the case of homologous recombination methods.	[78,79,80,81,82,83,84,85]
	KLF4	Suppresses EMT	Lentiviral vector			
	Id3	Sequestering bHLH transcription factors and preventing the downregulation of epithelial cell markers to hinder EMT.	pcDNA3-mCherry LIC mammalian expression vector construct			
	SMAD7	Prevents nuclear localization of SMAD2/3 and attenuates the TGF-β pathway by preventing the phosphorylation of SMAD3.	Recombinant adeno-associated viral vector			

Abbreviations: USP10: ubiquitin-specific protease-1; Kca3.1 ion channel: K+ channel 3.1 ion channel; KLF4: Krüppel-like factor 4; Id3: inhibitor of differentiation 3; SMAD7: mothers against decapentaplegic homolog 7.

**Table 3:** MicroRNAs as therapeutic tools in reviving a cornea without scarring.

Micro-RNA				Advantages	Limitation	Reference
Type	Level of Mirna During Healing Process	Therapeutic Regulatory Level Required	Mechanism of Action	Targeting several genes at once. Altering many signaling cascades at once.	No proper delivery vehicle for miRNA inside cell cytoplasm. Off-target silencing of genes.	
miR-204	Downregulated	Upregulation	Targets SMAD4			[87,88,89,90,91]
miR-145	Upregulated	Downregulation				[92,93,94,95]
miR-133b	Downregulated	Upregulation	Targets KLF4			[96,97,98]

Abbreviations: SMAD4: mothers against decapentaplegic homolog 4; KLF4: Krüppel-like factor 4.

**Table 4:** Biomolecules as therapeutic tools in reviving a cornea without scars.

Therapeutic Biomolecule	Therapeutic Strategy	Advantage	Limitation	Reference
Histone deacetylase inhibitors (e.g.,: SAHA)	Prevents deacetylation of H3 and H4 of anti-inflammatory genes, preventing their deactivation by histone deacetylases. Prevents deacetylation of SMAD7 by histone deacetylases.	Reversible epigenetic modification to create an antifibrotic environment.	HDAC inhibitors can have major side effects, such as reducing the number of viable T cells in the body and thrombocytopenia. Minor side effects include fatigue and nausea.	[100,101,102,103,104,105,106,107,108,109]
Growth factor (Insulin-like growth factor 1)	Converts keratocytes into collagenous keratocytes, which secrete native corneal ECM components.	Rules out myofibroblast formation and guides the cornea’s fibrotic healing process towards an antifibrotic method of wound healing.	Requires high doses. Growth factor can degrade inside the body, which indicates the need for a suitable delivery vehicle. Risk of antigenic response. Increased levels of one growth factor can influence other hormones. Not cost-effective.	[110,111,112]
Glucosamine	Increases the level of the phosphorylated form of PTEN (phosphatase), preventing dephosphorylation of KLF4 and increasing its stability to promote EMT. Post-translational modification of the proteasomal unit by n-acetyl glucosamine, reducing its proteasomal activity and increasing KLF4 stability.	Cost-effective, increasing KLF4 stability without the need for costly gene therapy.	Not suitable for diabetic patients. Increase the intraocular pressure and adverse effects in glaucoma patients.	[116,117,118,119,120]
Chitosan	Anti-angiogenic and antifibrotic properties.	Well-established wound-healing properties and highly biocompatible.	Chitosan nanoparticle preparation can be cumbersome.	[122,123,124,125,126]
Lycium barbarum polysaccharide (LBP)	Reduces profibrotic gene expression.	It is safe with no adverse side effects.	No standardization for the LBP concentration that must be used for efficient therapeutic effect. Lack of proper quality control for this herbal medicine.	[127,128,129]
JQ1	Inhibitor of BRD4; therefore, it does not allow the binding of BRD4 to keap1, and Nrf2 can remain in its stable form by binding to keap1. Nrf2 then translocates into the nucleus to increase antioxidant gene expression and decrease ROS levels.	Rules out the side effects of a conventional ocular drug, such as mitomycin C. Epigenetically modulate scarring without using an HDAC.	Bromodomain inhibitors have shown mixed results in clinical trials and some adverse side effects. May have a toxic effect on cells.	[136]
TPCA-1	An IKK inhibitor that attenuates the NF-κβ pathway.	Inhibiting NF-κβ pathway decreases the cytokine storm during the healing process.	Targets the NF-κβ pathway and not the TGF-β pathway, the key player in cornea fibrosis. Targeting NF-κβ can have severe adverse effects because it has both pro- and anti-inflammatory functions. It might attenuate the antifibrotic JAK/STAT pathway by STAT3 inhibition.	[137,138]
Acetylcholine	Promotes faster re-epithelization of the wounded corneal epithelial layer, activates protein kinase C, and decreases profibrotic gene expression.	Neurotransmitters can be structurally modified to obtain desired pharmacological activity.	There are chances that acetylcholine will be degraded by the cholinesterase of the eye. In vivo validation of the study is required. Lack of an efficient mode of delivery of acetylcholine into cornea.	[145,151]
Decorin	Activates CAM Kinase II to phosphorylate the serine 240 residue of SMAD2, forming the inhibitory SMAD2/3/4 complex, which cannot activate fibrotic genes.	A commonly used, clinically approved ocular drug to create an antifibrotic environment.	Poor retention time on the ocular surface.	[148,149,150]

Abbreviations: HDAC: histone deacetylase; SAHA: suberoylanilide hydroxamic acid; KLF4: Krüppel-like factor 4; Nrf2: nuclear factor erythroid 2–related factor 2; Keap1: kelch-like ECH-associated protein 1.

## References

[1] Song P., Wang S., Zhang P., Sui W., Zhang Y., Liu T., Gao H. (2016). The Superficial Stromal Scar Formation Mechanism in Keratoconus: A Study Using Laser Scanning In Vivo Confocal Microscopy. Biomed Res. Int..

[2] Wilson S.E., Netto M., Ambrósio R. (2003). Corneal Cells: Chatty in Development, Homeostasis, Wound Healing, and Disease. Am. J. Ophthalmol..

[3] West-Mays J.A., Dwivedi D.J. (2006). The Keratocyte: Corneal Stromal Cell with Variable Repair Phenotypes. Int. J. Biochem. Cell Biol..

[4] Whitcher J.P., Srinivasan M., Upadhyay M.P. (2001). Corneal Blindness: A Global Perspective. Bull. World Health Organ..

[5] Agrawal P.K. (1983). The pathology of cornea (a histopathological study). Indian J. Ophthalmol..

[6] McClintic S.M., Srinivasan M., Mascarenhas J., Greninger D.A., Acharya N.R., Lietman T.M., Keenan J.D. (2013). Improvement in Corneal Scarring Following Bacterial Keratitis. Eye.

[7] Gain P., Jullienne R., He Z., Aldossary M., Acquart S., Cognasse F., Thuret G. (2016). Global Survey of Corneal Transplantation and Eye Banking. JAMA Ophthalmol..

[8] Tseng S.C.G., Prabhasawat P., Lee S.-H. (1997). Amniotic Membrane Transplantation for Conjunctival Surface Reconstruction. Am. J. Ophthalmol..

[9] Salminen L. (1990). Review: Systemic Absorption of Topically Applied Ocular Drugs in Humans. J. Ocul. Pharmacol. Ther..

[10] Eghrari A.O., Riazuddin S.A., Gottsch J.D. (2015). Overview of the Cornea: Structure, Function, and Development. Progress in Molecular Biology and Translational Science.

[11] Mobaraki M., Abbasi R., Omidian Vandchali S., Ghaffari M., Moztarzadeh F., Mozafari M. (2019). Corneal Repair and Regeneration: Current Concepts and Future Directions. Front. Bioeng. Biotechnol..

[12] Wilson S.E. (2020). Bowman’s Layer in the Cornea– Structure and Function and Regeneration. Exp. Eye Res..

[13] Ljubimov A.V., Saghizadeh M. (2015). Progress in Corneal Wound Healing. Prog. Retin. Eye Res..

[14] Imanishi J., Kamiyama K., Iguchi I., Kita M., Sotozono C., Kinoshita S. (2000). Growth Factors: Importance in Wound Healing and Maintenance of Transparency of the Cornea. Prog. Retin. Eye Res..

[15] Haber M., Cao Z., Panjwani N., Bedenice D., Li W.W., Provost P.J. (2003). Effects of Growth Factors (EGF, PDGF-BB and TGF-Beta1) on Cultured Equine Epithelial Cells and Keratocytes: Implications for Wound Healing. Vet. Ophthalmol..

[16] Hong J.W., Liu J.J., Lee J.S., Mohan R.R., Mohan R.R., Woods D.J., He Y.G., Wilson S.E. (2001). Proinflammatory chemokine induction in keratocytes and inflammatory cell infiltration into the cornea. Investig. Ophthalmol. Vis. Sci..

[17] Stepp M.A. (2006). Corneal Integrins and Their Functions. Exp. Eye Res..

[18] Imai K., Hiramatsu A., Fukushima D., Pierschbacher M.D., Okada Y. (1997). Degradation of Decorin by Matrix Metalloproteinases: Identification of the Cleavage Sites, Kinetic Analyses and Transforming Growth Factor-Β1 Release. Biochem. J..

[19] Tandon A., Tovey J.C.K., Sharma A., Gupta R., Mohan R.R. (2010). Role of Transforming Growth Factor Beta in Corneal Function, Biology and Pathology. Curr. Mol. Med..

[20] Blanco-Mezquita J.T., Hutcheon A.E.K., Stepp M.A., Zieske J.D. (2011). AVβ6 Integrin Promotes Corneal Wound Healing. Investig. Opthalmol. Vis. Sci..

[21] Martin P., Leibovich S.J. (2005). Inflammatory Cells during Wound Repair: The Good, the Bad and the Ugly. Trends Cell Biol..

[22] Marrazzo G., Bellner L., Halilovic A., Li Volti G., Drago F., Dunn M.W., Schwartzman M.L. (2011). The Role of Neutrophils in Corneal Wound Healing in HO-2 Null Mice. PLoS ONE.

[23] Prame Kumar K., Nicholls A.J., Wong C.H.Y. (2018). Partners in Crime: Neutrophils and Monocytes/Macrophages in Inflammation and Disease. Cell Tissue Res..

[24] Chrysanthopoulou A., Mitroulis I., Apostolidou E., Arelaki S., Mikroulis D., Konstantinidis T., Sivridis E., Koffa M., Giatromanolaki A., Boumpas D.T. (2014). Neutrophil Extracellular Traps Promote Differentiation and Function of Fibroblasts. J. Pathol..

[25] Phan S.H. (2008). Biology of Fibroblasts and Myofibroblasts. Proc. Am. Thorac. Soc..

[26] Wilson S.E. (2012). Corneal Myofibroblast Biology and Pathobiology: Generation, Persistence, and Transparency. Exp. Eye Res..

[27] Huang T., David L., Mendoza V., Yang Y., Villarreal M., De K., Sun L., Fang X., López-Casillas F., Wrana J.L. (2011). TGF-β Signalling Is Mediated by Two Autonomously Functioning TβRI:TβRII Pairs. EMBO J..

[28] Walton K.L., Johnson K.E., Harrison C.A. (2017). Targeting TGF-β Mediated SMAD Signaling for the Prevention of Fibrosis. Front. Pharmacol..

[29] Słoniecka M., Danielson P. (2019). Substance P Induces Fibrotic Changes through Activation of the RhoA/ROCK Pathway in an in Vitro Human Corneal Fibrosis Model. J. Mol. Med..

[30] Prudnikova T.Y., Rawat S.J., Chernoff J. (2015). Molecular Pathways: Targeting the Kinase Effectors of RHO-Family GTPases. Clin. Cancer Res..

[31] Yu-Wai-Man C., Treisman R., Bailly M., Khaw P.T. (2014). The Role of the MRTF-A/SRF Pathway in Ocular Fibrosis. Investig. Opthalmol. Vis. Sci..

[32] Harvey S.A.K., Anderson S.C., SundarRaj N. (2004). Downstream Effects of ROCK Signaling in Cultured Human Corneal Stromal Cells: Microarray Analysis of Gene Expression. Investig. Opthalmol. Vis. Sci..

[33] Snijdelaar D.G., Dirksen R., Slappendel R., Crul B.J.P. (2000). Substance P. Eur. J. Pain.

[34] Suvas S. (2017). Role of Substance P Neuropeptide in Inflammation, Wound Healing, and Tissue Homeostasis. J. Immunol..

[35] Grgic I., Kiss E., Kaistha B.P., Busch C., Kloss M., Sautter J., Muller A., Kaistha A., Schmidt C., Raman G. (2009). Renal Fibrosis Is Attenuated by Targeted Disruption of KCa3.1 Potassium Channels. Proc. Natl. Acad. Sci. USA.

[36] Krstić J., Trivanović D., Mojsilović S., Santibanez J.F. (2015). Transforming Growth Factor-Beta and Oxidative Stress Interplay: Implications in Tumorigenesis and Cancer Progression. Oxid. Med. Cell. Longev..

[37] Chen L., Mongan M., Meng Q., Wang Q., Kao W., Xia Y. (2016). Corneal Wound Healing Requires IKB Kinase β Signaling in Keratocytes. PLoS ONE.

[38] Stratton M.S., Haldar S.M., McKinsey T.A. (2017). BRD4 Inhibition for the Treatment of Pathological Organ Fibrosis. F1000Research.

[39] Liu X.-F., Zhou D.-D., Xie T., Malik T.H., Lu C.-B., Li H.-J., Wang F., Shu C., Liu C., Lu C.-W. (2017). Nrf2, a Potential Therapeutic Target against Oxidative Stress in Corneal Diseases. Oxid. Med. Cell. Longev..

[40] Yuan J., Luo K., Zhang L., Cheville J.C., Lou Z. (2010). USP10 Regulates P53 Localization and Stability by Deubiquitinating P53. Cell.

[41] Steen E.H., Wang X., Balaji S., Butte M.J., Bollyky P.L., Keswani S.G. (2020). The Role of the Anti-Inflammatory Cytokine Interleukin-10 in Tissue Fibrosis. Adv. Wound Care.

[42] Ramaesh T., Ramaesh K., Leask R., Springbett A., Riley S.C., Dhillon B., West J.D. (2006). Increased Apoptosis and Abnormal Wound-Healing Responses in the Heterozygous *Pax6*^+/−^ Mouse Cornea. Investig. Opthalmol. Vis. Sci..

[43] Zheng K., Huang H., Peng K., Cai J., Jhanji V., Chen H. (2016). Change of Optical Intensity during Healing Process of Corneal Wound on Anterior Segment Optical Coherence Tomography. Sci. Rep..

[44] Ghatak S., Maytin E.V., Mack J.A., Hascall V.C., Atanelishvili I., Moreno Rodriguez R., Markwald R.R., Misra S. (2015). Roles of Proteoglycans and Glycosaminoglycans in Wound Healing and Fibrosis. Int. J. Cell Biol..

[45] Shu D.Y., Lovicu F.J. (2017). Myofibroblast Transdifferentiation: The Dark Force in Ocular Wound Healing and Fibrosis. Prog. Retin. Eye Res..

[46] Cintron C., Covington H.I., Kublin C.L. (1990). Morphologic Analyses of Proteoglycans in Rabbit Corneal Scars. Investig. Ophthalmol. Vis. Sci..

[47] Raposo G., Stoorvogel W. (2013). Extracellular Vesicles: Exosomes, Microvesicles, and Friends. J. Cell Biol..

[48] Turturici G., Tinnirello R., Sconzo G., Geraci F. (2014). Extracellular Membrane Vesicles as a Mechanism of Cell-to-Cell Communication: Advantages and Disadvantages. Am. J. Physiol. Physiol..

[49] Das C.K., Jena B.C., Banerjee I., Das S., Parekh A., Bhutia S.K., Mandal M. (2019). Exosome as a Novel Shuttle for Delivery of Therapeutics across Biological Barriers. Mol. Pharm..

[50] Kuschert G.S.V., Hoogewerf A.J., Proudfoot A.E.I., Chung C., Cooke R.M., Hubbard R.E., Wells T.N.C., Sanderson P.N. (1998). Identification of a Glycosaminoglycan Binding Surface on Human Interleukin-8. Biochemistry.

[51] Hertsenberg A.J., Shojaati G., Funderburgh M.L., Mann M.M., Du Y., Funderburgh J.L. (2017). Corneal Stromal Stem Cells Reduce Corneal Scarring by Mediating Neutrophil Infiltration after Wounding. PLoS ONE.

[52] Dyer D.P., Thomson J.M., Hermant A., Jowitt T.A., Handel T.M., Proudfoot A.E.I., Day A.J., Milner C.M. (2014). TSG-6 Inhibits Neutrophil Migration via Direct Interaction with the Chemokine CXCL8. J. Immunol..

[53] Shojaati G., Khandaker I., Funderburgh M.L., Mann M.M., Basu R., Stolz D.B., Geary M.L., Dos Santos A., Deng S.X., Funderburgh J.L. (2019). Mesenchymal Stem Cells Reduce Corneal Fibrosis and Inflammation via Extracellular Vesicle-Mediated Delivery of MiRNA. Stem Cells Transl. Med..

[54] Shen T., Zheng Q.-Q., Shen J., Li Q.-S., Song X.-H., Luo H.-B., Hong C.-Y., Yao K. (2018). Effects of Adipose-Derived Mesenchymal Stem Cell Exosomes on Corneal Stromal Fibroblast Viability and Extracellular Matrix Synthesis. Chin. Med. J. (Engl)..

[55] Hofmann T.G., Stollberg N., Schmitz M.L., Will H. (2003). HIPK2 Regulates Transforming Growth Factor-Beta-Induced c-Jun NH(2)-Terminal Kinase Activation and Apoptosis in Human Hepatoma Cells. Cancer Res..

[56] Shen T., Zheng Q., Luo H., Li X., Chen Z., Song Z., Zhou G., Hong C. (2020). Exosomal MiR-19a from Adipose-Derived Stem Cells Suppresses Differentiation of Corneal Keratocytes into Myofibroblasts. Aging (Albany NY).

[57] Lai Y., Lee P., Lu C., Liu Y., Wang S., Liu C., Chang Y., Chen Y., Su C., Li C. (2021). Thrombospondin 1-induced Exosomal Proteins Attenuate Hypoxia-induced Paraptosis in Corneal Epithelial Cells and Promote Wound Healing. FASEB J..

[58] Tao H., Chen X., Cao H., Zheng L., Li Q., Zhang K., Han Z., Han Z.-C., Guo Z., Li Z. (2019). Mesenchymal Stem Cell-Derived Extracellular Vesicles for Corneal Wound Repair. Stem Cells Int..

[59] Jablonska E., Garley M., Surazynski A., Grubczak K., Iwaniuk A., Borys J., Moniuszko M., Ratajczak-Wrona W. (2020). Neutrophil Extracellular Traps (NETs) Formation Induced by TGF-β in Oral Lichen Planus—Possible Implications for the Development of Oral Cancer. Immunobiology.

[60] Mun Y., Hwang J.S., Shin Y.J. (2021). Role of Neutrophils on the Ocular Surface. Int. J. Mol. Sci..

[61] Yin H., Lu Q., Wang X., Majumdar S., Jun A.S., Stark W.J., Grant M.P., Elisseeff J.H. (2019). Tissue-Derived Microparticles Reduce Inflammation and Fibrosis in Cornea Wounds. Acta Biomater..

[62] Yamaguchi T. (2018). Inflammatory Response in Dry Eye. Investig. Opthalmol. Vis. Sci..

[63] Peterson J.L., Ceresa B.P. (2021). Epidermal Growth Factor Receptor Expression in the Corneal Epithelium. Cells.

[64] Alto L.T., Terman J.R., Terman J.R. (2017). Semaphorins and Their Signaling Mechanisms. Semaphorin Signaling, Methods in Molecular Biology.

[65] Jeon K.-I., Nehrke K., Huxlin K.R. (2020). Semaphorin 3A Potentiates the Profibrotic Effects of Transforming Growth Factor-Β1 in the Cornea. Biochem. Biophys. Res. Commun..

[66] Morishige N., Ko J.-A., Morita Y., Nishida T. (2010). Expression of Semaphorin 3A in the Rat Corneal Epithelium during Wound Healing. Biochem. Biophys. Res. Commun..

[67] Yamazaki R., Yamazoe K., Yoshida S., Hatou S., Inagaki E., Okano H., Tsubota K., Shimmura S. (2017). The Semaphorin 3A Inhibitor SM-345431 Preserves Corneal Nerve and Epithelial Integrity in a Murine Dry Eye Model. Sci. Rep..

[68] Takayama K., Suzuki T., Fujimura T., Takahashi S., Inoue S. (2018). Association of USP10 with G3BP2 Inhibits P53 Signaling and Contributes to Poor Outcome in Prostate Cancer. Mol. Cancer Res..

[69] Zhang H., Zhang S., He H., Zhang C., Yu D., Shao R. (2013). Downregulation of G3BPs Inhibits the Growth, Migration and Invasion of Human Lung Carcinoma H1299 Cells by Suppressing the Src/FAK-Associated Signaling Pathway. Cancer Gene Ther..

[70] Gillespie S.R., Tedesco L.J., Wang L., Bernstein A.M. (2017). The Deubiquitinase USP10 Regulates Integrin Beta1 and Beta5 and Fibrotic Wound Healing. J. Cell Sci..

[71] Boumil E.F., Castro N., Phillips A.T., Chatterton J.E., McCauley S.M., Wolfson A.D., Shmushkovich T., Ridilla M., Bernstein A.M. (2020). USP10 Targeted Self-Deliverable SiRNA to Prevent Scarring in the Cornea. Mol. Ther. Nucleic Acids.

[72] Morales P., Garneau L., Klein H., Lavoie M.-F., Parent L., Sauvé R. (2013). Contribution of the KCa3.1 Channel–Calmodulin Interactions to the Regulation of the KCa3.1 Gating Process. J. Gen. Physiol..

[73] Pérez-García M.T., Cidad P., López-López J.R. (2018). The Secret Life of Ion Channels: Kv1.3 Potassium Channels and Proliferation. Am. J. Physiol. Physiol..

[74] Girault A., Chebli J., Privé A., Trinh N.T.N., Maillé E., Grygorczyk R., Brochiero E. (2015). Complementary Roles of KCa3.1 Channels and Β1-Integrin during Alveolar Epithelial Repair. Respir. Res..

[75] Arcangeli A., Becchetti A. (2006). Complex Functional Interaction between Integrin Receptors and Ion Channels. Trends Cell Biol..

[76] Takada Y., Ye X., Simon S. (2007). The integrins. Genome. Biol..

[77] Brown B.M., Pressley B., Wulff H. (2018). KCa3.1 Channel Modulators as Potential Therapeutic Compounds for Glioblastoma. Curr. Neuropharmacol..

[78] Anumanthan G., Gupta S., Fink M.K., Hesemann N.P., Bowles D.K., McDaniel L.M., Muhammad M., Mohan R.R. (2018). KCa3.1 Ion Channel: A Novel Therapeutic Target for Corneal Fibrosis. PLoS ONE.

[79] Fujimoto S., Hayashi R., Hara S., Sasamoto Y., Harrington J., Tsujikawa M., Nishida K. (2019). KLF4 Prevents Epithelial to Mesenchymal Transition in Human Corneal Epithelial Cells via Endogenous TGF-Β2 Suppression. Regen. Ther..

[80] Gupta S., Martin L.M., Sinha N.R., Smith K.E., Sinha P.R., Dailey E.M., Hesemann N.P., Mohan R.R. (2020). Role of Inhibitor of Differentiation 3 Gene in Cellular Differentiation of Human Corneal Stromal Fibroblasts. Mol. Vis..

[81] Saitoh M., Miyazawa K. (2012). Transcriptional and Post-Transcriptional Regulation in TGF—Mediated Epithelial-Mesenchymal Transition. J. Biochem..

[82] Xu J., Lamouille S., Derynck R. (2009). TGF-β-Induced Epithelial to Mesenchymal Transition. Cell Res..

[83] Chaudhary J., Sadler-Riggleman I., Ague J.M., Skinner M.K. (2005). The Helix-Loop-Helix Inhibitor of Differentiation (ID) Proteins Induce Post-Mitotic Terminally Differentiated Sertoli Cells to Re-Enter the Cell Cycle and Proliferate. Biol. Reprod..

[84] Yan X., Liao H., Cheng M., Shi X., Lin X., Feng X.-H., Chen Y.-G. (2016). Smad7 Protein Interacts with Receptor-Regulated Smads (R-Smads) to Inhibit Transforming Growth Factor-β (TGF-β)/Smad Signaling. J. Biol. Chem..

[85] Kavsak P., Rasmussen R.K., Causing C.G., Bonni S., Zhu H., Thomsen G.H., Wrana J.L. (2000). Smad7 Binds to Smurf2 to Form an E3 Ubiquitin Ligase That Targets the TGFβ Receptor for Degradation. Mol. Cell.

[86] Gupta S., Rodier J.T., Sharma A., Giuliano E.A., Sinha P.R., Hesemann N.P., Ghosh A., Mohan R.R. (2017). Targeted AAV5-Smad7 Gene Therapy Inhibits Corneal Scarring In Vivo. PLoS ONE.

[87] O’Brien J., Hayder H., Zayed Y., Peng C. (2018). Overview of MicroRNA Biogenesis, Mechanisms of Actions, and Circulation. Front. Endocrinol. (Lausanne).

[88] An J., Chen X., Chen W., Liang R., Reinach P.S., Yan D., Tu L. (2015). MicroRNA Expression Profile and the Role of MiR-204 in Corneal Wound Healing. Investig. Opthalmol. Vis. Sci..

[89] Wang Y., Li W., Zang X., Chen N., Liu T., Tsonis P.A., Huang Y. (2013). MicroRNA-204-5p Regulates Epithelial-to-Mesenchymal Transition during Human Posterior Capsule Opacification by Targeting SMAD4. Investig. Opthalmol. Vis. Sci..

[90] Li Y., Zhao Z., Xu C., Zhou Z., Zhu Z., You T. (2014). HMGA2 Induces Transcription Factor Slug Expression to Promote Epithelial-to-Mesenchymal Transition and Contributes to Colon Cancer Progression. Cancer Lett..

[91] Medici D., Hay E.D., Olsen B.R. (2008). Snail and Slug Promote Epithelial-Mesenchymal Transition through β-Catenin–T-Cell Factor-4-Dependent Expression of Transforming Growth Factor-Β3. Mol. Biol. Cell.

[92] Lu Y., Tai P.W.L., Ai J., Gessler D.J., Su Q., Yao X., Zheng Q., Zamore P.D., Xu X., Gao G. (2018). Transcriptome Profiling of Neovascularized Corneas Reveals MiR-204 as a Multi-Target Biotherapy Deliverable by RAAVs. Mol. Ther. Nucleic Acids.

[93] Ratuszny D., Gras C., Bajor A., Börger A.-K., Pielen A., Börgel M., Framme C., Blasczyk R., Figueiredo C. (2015). MiR-145 Is a Promising Therapeutic Target to Prevent Cornea Scarring. Hum. Gene Ther..

[94] Xu N., Papagiannakopoulos T., Pan G., Thomson J.A., Kosik K.S. (2009). MicroRNA-145 Regulates OCT4, SOX2, and KLF4 and Represses Pluripotency in Human Embryonic Stem Cells. Cell.

[95] Sun H., Peng Z., Tang H., Xie D., Jia Z., Zhong L., Zhao S., Ma Z., Gao Y., Zeng L. (2017). Loss of KLF4 and Consequential Downregulation of Smad7 Exacerbate Oncogenic TGF-β Signaling in and Promote Progression of Hepatocellular Carcinoma. Oncogene.

[96] Li X.M., Kim S.J., Hong D.-K., Jung K.E., Choi C.W., Seo Y.-J., Lee J.-H., Lee Y., Kim C.-D. (2019). KLF4 Suppresses the Tumor Activity of Cutaneous Squamous Cell Carcinoma (SCC) Cells via the Regulation of SMAD Signaling and SOX2 Expression. Biochem. Biophys. Res. Commun..

[97] Gjymishka A., Pi L., Oh S.-H., Jorgensen M., Liu C., Protopapadakis Y., Patel A., Petersen B.E. (2016). MiR-133b Regulation of Connective Tissue Growth Factor. Am. J. Pathol..

[98] Grotendorst G.R., Duncan M.R. (2005). Individual Domains of Connective Tissue Growth Factor Regulate Fibroblast Proliferation and Myofibroblast Differentiation. FASEB J..

[99] Zhao X., Song W., Chen Y., Liu S., Ren L. (2019). Collagen-Based Materials Combined with MicroRNA for Repairing Cornea Wounds and Inhibiting Scar Formation. Biomater. Sci..

[100] Ashby B.D., Garrett Q., Willcox M.D.P. (2014). Corneal Injuries and Wound Healing—Review of Processes and Therapies. Austin J. Clin. Ophthalmol..

[101] Landén N.X., Li D., Ståhle M. (2016). Transition from Inflammation to Proliferation: A Critical Step during Wound Healing. Cell. Mol. Life Sci..

[102] Zhou Q., Wang Y., Yang L., Wang Y., Chen P., Wang Y., Dong X., Xie L. (2008). Histone Deacetylase Inhibitors Blocked Activation and Caused Senescence of Corneal Stromal cells. Mol. Vis..

[103] Sun G., Reddy M.A., Yuan H., Lanting L., Kato M., Natarajan R. (2010). Epigenetic Histone Methylation Modulates Fibrotic Gene Expression. J. Am. Soc. Nephrol..

[104] Lu C., Sidoli S., Kulej K., Ross K., Wu C.H., Garcia B.A. (2019). Coordination between TGF-β Cellular Signaling and Epigenetic Regulation during Epithelial to Mesenchymal Transition. Epigenetics Chromatin.

[105] Tang J., Yan H., Zhuang S. (2013). Histone Deacetylases as Targets for Treatment of Multiple Diseases. Clin. Sci..

[106] Zhou Q., Yang L., Wang Y., Qu M., Chen P., Wang Y., Xie L., Zhao J., Wang Y. (2010). TGFβ Mediated Transition of Corneal Fibroblasts from a Proinflammatory State to a Profibrotic State through Modulation of Histone Acetylation. J. Cell. Physiol..

[107] Xiao W., Chen X., Liu X., Luo L., Ye S., Liu Y. (2014). Trichostatin A, a Histone Deacetylase Inhibitor, Suppresses Proliferation and Epithelial–Mesenchymal Transition in Retinal Pigment Epithelium Cells. J. Cell. Mol. Med..

[108] Sharma A., Mehan M.M., Sinha S., Cowden J.W., Mohan R.R. (2009). Trichostatin A Inhibits Corneal Haze In Vitro and In Vivo. Investig. Opthalmol. Vis. Sci..

[109] Donnelly K.S., Giuliano E.A., Sharma A., Mohan R.R. (2014). Suberoylanilide Hydroxamic Acid (Vorinostat): Its Role on Equine Corneal Fibrosis and Matrix Metalloproteinase Activity. Vet. Ophthalmol..

[110] Etheredge L., Kane B.P., Hassell J.R. (2009). The Effect of Growth Factor Signaling on Keratocytes In Vitro and Its Relationship to the Phases of Stromal Wound Repair. Investig. Opthalmol. Vis. Sci..

[111] Sarenac T., Trapecar M., Gradisnik L., Rupnik M.S., Pahor D. (2016). Single-Cell Analysis Reveals IGF-1 Potentiation of Inhibition of the TGF-β/Smad Pathway of Fibrosis in Human Keratocytes In Vitro. Sci. Rep..

[112] Ghiasi Z., Gray T., Tran P., Dubielzig R., Murphy C., McCartney D.L., Reid T.W. (2018). The Effect of Topical Substance-P Plus Insulin-like Growth Factor-1 (IGF-1) on Epithelial Healing After Photorefractive Keratectomy in Rabbits. Transl. Vis. Sci. Technol..

[113] Chen Y.-J., Huang Y.-S., Chen J.-T., Chen Y.-H., Tai M.-C., Chen C.-L., Liang C.-M. (2015). Protective Effects of Glucosamine on Oxidative-Stress and Ischemia/Reperfusion-Induced Retinal Injury. Investig. Ophthalmol. Vis. Sci..

[114] Pham T., Cornea A., Jenkins A., Blick K.E., Scofield R.H. (2007). Oral Glucosamine in Doses Used to Treat Osteoarthritis Worsens Insulin Resistance. Am. J. Med. Sci..

[115] Esfandiari H., Loewen N.A. (2019). Effect of Glucosamine on Intraocular Pressure. Handbook of Nutrition, Diet, and the Eye.

[116] Park J., Lee S.-Y., Ooshima A., Yang K.-M., Kang J.M., Kim Y.-W., Kim S.-J. (2013). Glucosamine Hydrochloride Exerts a Protective Effect against Unilateral Ureteral Obstruction-Induced Renal Fibrosis by Attenuating TGF-β Signaling. J. Mol. Med..

[117] Wang D.-F., Yang H.-J., GU J.-Q., Cao Y.-L., Meng X., Wang X.-L., Lin Y.-C., Gao M. (2015). Suppression of Phosphatase and Tensin Homolog Protects Insulin-Resistant Cells from Apoptosis. Mol. Med. Rep..

[118] Li H., Han M., Bernier M., Zheng B., Sun S., Su M., Zhang R., Fu J., Wen J. (2010). Krüppel-like Factor 4 Promotes Differentiation by Transforming Growth Factor-β Receptor-Mediated Smad and P38 MAPK Signaling in Vascular Smooth Muscle Cells. J. Biol. Chem..

[119] He M., Zheng B., Zhang Y., Zhang X.-H., Wang C., Yang Z., Sun Y., Wu X.-L., Wen J.-K. (2015). KLF4 Mediates the Link between TGF-Β1-Induced Gene Transcription and H3 Acetylation in Vascular Smooth Muscle Cells. FASEB J..

[120] Zhang F., Su K., Yang X., Bowe D.B., Paterson A.J., Kudlow J.E. (2003). O-GlcNAc Modification Is an Endogenous Inhibitor of the Proteasome. Cell.

[121] Swamynathan S., Buela K.-A., Kinchington P., Lathrop K.L., Misawa H., Hendricks R.L., Swamynathan S.K. (2012). Klf4 Regulates the Expression of Slurp1, Which Functions as an Immunomodulatory Peptide in the Mouse Cornea. Investig. Opthalmol. Vis. Sci..

[122] Azad A.K., Sermsintham N., Chandrkrachang S., Stevens W.F. (2004). Chitosan Membrane as a Wound-Healing Dressing: Characterization and Clinical Application. J. Biomed. Mater. Res..

[123] Alsarra I.A. (2009). Chitosan Topical Gel Formulation in the Management of Burn Wounds. Int. J. Biol. Macromol..

[124] Fischak C., Klaus R., Werkmeister R.M., Hohenadl C., Prinz M., Schmetterer L., Garhöfer G. (2017). Effect of Topically Administered Chitosan *N* Acetylcysteine on Corneal Wound Healing in a Rabbit Model. J. Ophthalmol..

[125] Zahir-Jouzdani F., Mahbod M., Soleimani M., Vakhshiteh F., Arefian E., Shahosseini S., Dinarvand R., Atyabi F. (2018). Chitosan and Thiolated Chitosan: Novel Therapeutic Approach for Preventing Corneal Haze after Chemical Injuries. Carbohydr. Polym..

[126] Chen Q., Lu H., Yang H. (2014). Chitosan Inhibits Fibroblasts Growth in Achilles Tendon via TGF-Β1/Smad3 Pathway by MiR-29b. Int. J. Clin. Exp. Pathol..

[127] Gan F., Liu Q., Liu Y., Huang D., Pan C., Song S., Huang K. (2018). Lycium Barbarum Polysaccharides Improve CCl4-Induced Liver Fibrosis, Inflammatory Response and TLRs/NF-KB Signaling Pathway Expression in Wistar Rats. Life Sci..

[128] Du S., Han B., Li K., Zhang X., Sha X., Gao L. (2017). *Lycium Barbarum* Polysaccharides Protect Rat Corneal Epithelial Cells against Ultraviolet B-Induced Apoptosis by Attenuating the Mitochondrial Pathway and Inhibiting JNK Phosphorylation. Biomed Res. Int..

[129] Kwok S.S., Wong F.S.-Y., Shih K.C., Chan Y.-K., Bu Y., Chan T.C.-Y., Ng A.L.-K., Lo A.C.-Y., Tong L., Yam G.H.-F. (2020). Lycium Barbarum Polysaccharide Suppresses Expression of Fibrotic Proteins in Primary Human Corneal Fibroblasts. J. Clin. Med..

[130] Richter K., Konzack A., Pihlajaniemi T., Heljasvaara R., Kietzmann T. (2015). Redox-Fibrosis: Impact of TGFβ1 on ROS Generators, Mediators and Functional Consequences. Redox Biol..

[131] Cucoranu I., Clempus R., Dikalova A., Phelan P.J., Ariyan S., Dikalov S., Sorescu D. (2005). NAD(P)H Oxidase 4 Mediates Transforming Growth Factor-Β1–Induced Differentiation of Cardiac Fibroblasts Into Myofibroblasts. Circ. Res..

[132] Liu R.-M., Desai L.P. (2015). Reciprocal Regulation of TGF-β and Reactive Oxygen Species: A Perverse Cycle for Fibrosis. Redox Biol..

[133] Barnes J.L., Gorin Y. (2011). Myofibroblast Differentiation during Fibrosis: Role of NAD(P)H Oxidases. Kidney Int..

[134] Bourji K., Meyer A., Chatelus E., Pincemail J., Pigatto E., Defraigne J.-O., Singh F., Charlier C., Geny B., Gottenberg J.-E. (2015). High Reactive Oxygen Species in Fibrotic and Nonfibrotic Skin of Patients with Diffuse Cutaneous Systemic Sclerosis. Free Radic. Biol. Med..

[135] Serafini M.M., Catanzaro M., Fagiani F., Simoni E., Caporaso R., Dacrema M., Romanoni I., Govoni S., Racchi M., Daglia M. (2020). Modulation of Keap1/Nrf2/ARE Signaling Pathway by Curcuma- and Garlic-Derived Hybrids. Front. Pharmacol..

[136] Qu M., Zhang X., Hu X., Dong M., Pan X., Bian J., Zhou Q. (2018). BRD4 Inhibitor JQ1 Inhibits and Reverses Mechanical Injury-Induced Corneal Scarring. Cell Death Discov..

[137] Balser C., Wolf A., Herb M., Langmann T. (2019). Co-Inhibition of PGF and VEGF Blocks Their Expression in Mononuclear Phagocytes and Limits Neovascularization and Leakage in the Murine Retina. J. Neuroinflamm..

[138] Zhang W., Chen J., Qu M., Backman L.J., Zhang A., Liu H., Zhang X., Zhou Q., Danielson P. (2020). Sustained Release of TPCA-1 from Silk Fibroin Hydrogels Preserves Keratocyte Phenotype and Promotes Corneal Regeneration by Inhibiting Interleukin-1 *β* Signaling. Adv. Healthc. Mater..

[139] Kondo Y., Fukuda K., Adachi T., Nishida T. (2008). Inhibition by a Selective IκB Kinase-2 Inhibitor of Interleukin-1–Induced Collagen Degradation by Corneal Fibroblasts in Three-Dimensional Culture. Investig. Opthalmol. Vis. Sci..

[140] Mulholland B., Tuft S.J., Khaw P.T. (2005). Matrix Metalloproteinase Distribution during Early Corneal Wound Healing. Eye.

[141] Pardo A., Cabrera S., Maldonado M., Selman M. (2016). Role of Matrix Metalloproteinases in the Pathogenesis of Idiopathic Pulmonary Fibrosis. Respir. Res..

[142] Stevenson R.W., Wilson W.S. (1974). Drug-Induced Depletion of Acetylcholine in the Rabbit Corneal Epithelium. Biochem. Pharmacol..

[143] Chernyavsky A.I., Galitovskiy V., Shchepotin I.B., Jester J.V., Grando S.A. (2014). The Acetylcholine Signaling Network of Corneal Epithelium and Its Role in Regulation of Random and Directional Migration of Corneal Epithelial Cells. Investig. Ophthalmol. Vis. Sci..

[144] Öztürk F., Kurt E., Inan Ü.Ü., Emiroglu L., Ilker S.S. (1999). The Effects of Acetylcholine and Propolis Extract on Corneal Epithelial Wound Healing in Rats. Cornea.

[145] Słoniecka M., Backman L.J., Danielson P. (2015). Acetylcholine Enhances Keratocyte Proliferation through Muscarinic Receptor Activation. Int. Immunopharmacol..

[146] Gubbiotti M.A., Vallet S.D., Ricard-Blum S., Iozzo R.V. (2016). Decorin Interacting Network: A Comprehensive Analysis of Decorin-Binding Partners and Their Versatile Functions. Matrix Biol..

[147] Mohan R.R., Gupta R., Mehan M.K., Cowden J.W., Sinha S. (2010). Decorin Transfection Suppresses Profibrogenic Genes and Myofibroblast Formation in Human Corneal Fibroblasts. Exp. Eye Res..

[148] ABDEL-WAHAB N., WICKS S.J., MASON R.M., CHANTRY A. (2002). Decorin Suppresses Transforming Growth Factor-β-Induced Expression of Plasminogen Activator Inhibitor-1 in Human Mesangial Cells through a Mechanism That Involves Ca2+-Dependent Phosphorylation of Smad2 at Serine-240. Biochem. J..

[149] Chouhan G., Moakes R.J.A., Esmaeili M., Hill L.J., deCogan F., Hardwicke J., Rauz S., Logan A., Grover L.M. (2019). A Self-Healing Hydrogel Eye Drop for the Sustained Delivery of Decorin to Prevent Corneal Scarring. Biomaterials.

[150] Hill L.J., Moakes R.J.A., Vareechon C., Butt G., Ng A., Brock K., Chouhan G., Vincent R.C., Abbondante S., Williams R. (2018). Sustained release of decorin to the surface of the eye enables scarless corneal regeneration. npj Regen. Med..

[151] Słoniecka M., Danielson P. (2020). Acetylcholine Decreases Formation of Myofibroblasts and Excessive Extracellular Matrix Production in an in Vitro Human Corneal Fibrosis Model. J. Cell. Mol. Med..

[152] Chaurasia S., Lim R., Lakshminarayanan R., Mohan R. (2015). Nanomedicine Approaches for Corneal Diseases. J. Funct. Biomater..

[153] Pissuwan D., Niidome T., Cortie M.B. (2011). The Forthcoming Applications of Gold Nanoparticles in Drug and Gene Delivery Systems. J. Control. Release.

[154] Tandon A., Sharma A., Rodier J.T., Klibanov A.M., Rieger F.G., Mohan R.R. (2013). BMP7 Gene Transfer via Gold Nanoparticles into Stroma Inhibits Corneal Fibrosis In Vivo. PLoS ONE.

[155] Bhatta R.S., Chandasana H., Chhonker Y.S., Rathi C., Kumar D., Mitra K., Shukla P.K. (2012). Mucoadhesive Nanoparticles for Prolonged Ocular Delivery of Natamycin: In Vitro and Pharmacokinetics Studies. Int. J. Pharm..

[156] Sharma A., Tandon A., Tovey J.C.K., Gupta R., Robertson J.D., Fortune J.A., Klibanov A.M., Cowden J.W., Rieger F.G., Mohan R.R. (2011). Polyethylenimine-Conjugated Gold Nanoparticles: Gene Transfer Potential and Low Toxicity in the Cornea. Nanomed. Nanotechnol. Biol. Med..

[157] Sriram S., Robinson P., Pi L., Lewin A.S., Schultz G. (2013). Triple Combination of SiRNAs Targeting TGFβ1, TGFβR2, and CTGF Enhances Reduction of Collagen I and Smooth Muscle Actin in Corneal Fibroblasts. Investig. Opthalmol. Vis. Sci..

[158] Zahir-Jouzdani F., Soleimani M., Mahbod M., Mottaghitalab F., Vakhshite F., Arefian E., Shahhoseini S., Dinarvand R., Atyabi F. (2018). Corneal Chemical Burn Treatment through a Delivery System Consisting of TGF-Β1 SiRNA: In Vitro and In Vivo. Drug Deliv. Transl. Res..

[159] Silva R.O., da Costa B.L., da Silva F.R., da Silva C.N., de Paiva M.B., Dourado L.F.N., Malachias A., de Souza Araújo A.A., Nunes P.S., Silva-Cunha A. (2019). Treatment for Chemical Burning Using Liquid Crystalline Nanoparticles as an Ophthalmic Delivery System for Pirfenidone. Int. J. Pharm..

[160] Kramerov A.A., Shah R., Ding H., Holler E., Turjman S., Rabinowitz Y.S., Ghiam S., Maguen E., Svendsen C.N., Saghizadeh M. (2021). Novel Nanopolymer RNA Therapeutics Normalize Human Diabetic Corneal Wound Healing and Epithelial Stem Cells. Nanomed. Nanotechnol. Biol. Med..

[161] Ma X.-Y., Bao H.-J., Cui L., Zou J. (2013). The Graft of Autologous Adipose-Derived Stem Cells in the Corneal Stromal after Mechanic Damage. PLoS ONE.

[162] Cejkova J., Cejka C., Trosan P., Zajicova A., Sykova E., Holan V. (2016). Treatment of Alkali-Injured Cornea by Cyclosporine A-Loaded Electrospun Nanofibers—An Alternative Mode of Therapy. Exp. Eye Res..

[163] Duan X., Sheardown H. (2006). Dendrimer Crosslinked Collagen as a Corneal Tissue Engineering Scaffold: Mechanical Properties and Corneal Epithelial Cell Interactions. Biomaterials.

[164] Ibrahim Al-Mashahedah A.M., Kanwar R.K., Kanwar J.R. (2019). Utility of Nanomedicine Targeting Scar-Forming Myofibroblasts to Attenuate Corneal Scarring and Haze. Nanomedicine.

[165] Swaminathan S., Vavia P.R., Trotta F., Cavalli R. (2013). Nanosponges Encapsulating Dexamethasone for Ocular Delivery: Formulation Design, Physicochemical Characterization, Safety and Corneal Permeability Assessment. J. Biomed. Nanotechnol..

[166] Weng Y., Liu J., Jin S., Guo W., Liang X., Hu Z. (2017). Nanotechnology-Based Strategies for Treatment of Ocular Disease. Acta Pharm. Sin. B.

[167] Akbari A., Jabbari N., Sharifi R., Ahmadi M., Vahhabi A., Seyedzadeh S.J., Nawaz M., Szafert S., Mahmoodi M., Jabbari E. (2020). Free and Hydrogel Encapsulated Exosome-Based Therapies in Regenerative Medicine. Life Sci..

[168] Isaacson A., Swioklo S., Connon C.J. (2018). 3D Bioprinting of a Corneal Stroma Equivalent. Exp. Eye Res..

[169] Kim H., Jang J., Park J., Lee K.-P., Lee S., Lee D.-M., Kim K.H., Kim H.K., Cho D.-W. (2019). Shear-Induced Alignment of Collagen Fibrils Using 3D Cell Printing for Corneal Stroma Tissue Engineering. Biofabrication.

[170] Duarte Campos D.F., Rohde M., Ross M., Anvari P., Blaeser A., Vogt M., Panfil C., Yam G.H., Mehta J.S., Fischer H. (2019). Corneal Bioprinting Utilizing Collagen-based Bioinks and Primary Human Keratocytes. J. Biomed. Mater. Res. Part A.

